# circACTA2 inhibits NLRP3 inflammasome-mediated inflammation via interacting with NF-κB in vascular smooth muscle cells

**DOI:** 10.1007/s00018-023-04840-6

**Published:** 2023-07-27

**Authors:** Yang Bai, Long Zhang, Bin Zheng, Xinhua Zhang, Hong Zhang, Anning Zhao, Jing Yu, Zhan Yang, Jinkun Wen

**Affiliations:** 1 Department of Biochemistry and Molecular Biology, The Key Laboratory of Neural and Vascular Biology, Ministry of Education of China, Hebei Medical University, 361 Zhongshan East Road, Shijiazhuang, 050017 China; 2 Molecular Biology Laboratory, Talent and Academic Exchange Center, The Second Hospital of Hebei Medical University, 215 Heping West Road, Shijiazhuang, 050017 China; 3 Department of Urology, The Second Hospital of Hebei Medical University, 215 Heping West Road, Shijiazhuang, 050017 China; 4 Department of Respiratory, The Second Hospital of Hebei Medical University, 215 Heping West Road, Shijiazhuang, 050017 China; 5 Institution of Chinese Integrative Medicine, Hebei Medical University, 361 Zhongshan East Road, Shijiazhuang, 050017 China

**Keywords:** circACTA2, NLRP3, NF-κB, VSMC, Inflammation, Vascular remodeling

## Abstract

circACTA2 derived from the smooth muscle α-actin gene plays an important role in the regulation of vascular smooth muscle cell (VSMC) phenotype. The activation of NLRP3 inflammasome is involved in VSMC phenotypic switching. However, the mechanistic relationship between circACTA2 and NLRP3 inflammasome during vascular remodeling remains poorly understood. Here, we showed that circACTA2 was down-regulated in human intimal hyperplasia. circACTA2 overexpression in circACTA2 transgenic mice significantly decreased the neointimal hyperplasia induced by vascular injury, which is concomitant with a decrease in IL-18, IL-1β, TNF-α, and IL-6 levels. Gain- and loss-of-function studies revealed that circACTA2 alleviated VSMC inflammation by suppressing the activation of NLRP3 inflammasome. Mechanistically, circACTA2 inhibited the expression of NF-κB p65 and p50 subunits and interacted with p50, which impedes the formation of the p50/p65 heterodimer and nuclear translocation induced by TNF-α, thus resulting in the suppression of NLRP3 gene transcription and inflammasome activation. Furthermore, circACTA2 overexpression mitigated inflammation via repressing NLRP3 inflammasome-mediated VSMC pyroptosis. Importantly, employing a decoy oligonucleotide to compete with circACTA2 for binding to p50 could attenuate the expression of NLRP3, ASC, and caspase-1. These findings provide a novel insight into the functional roles of circACTA2 in VSMCs, and targeting the circACTA2–NF-κB–NLRP3 axis represents a promising therapeutic strategy for vascular remodeling.

**Supplementary Information:**

The online version contains supplementary material available at 10.1007/s00018-023-04840-6.

## Introduction

Vascular remodeling is a common pathological feature of multiple cardiovascular diseases, such as hypertension, atherosclerosis, aneurysm, and restenosis after angioplasty, which involves VSMC proliferation, migration, and extracellular matrix remodeling caused by inflammation and oxidative stress in blood vessels [1–3]. Vascular smooth muscle cells (VSMCs) are located in the medial layer of all the blood vessels, where they are maintained in a more differentiated contractile state and are responsible for arterial contraction and production of extracellular matrix (ECM) [4]. In response to vascular injury, VSMCs switch to a synthetic phenotype and contribute to vascular remodeling or neointimal hyperplasia [5]. Increasing evidence has shown that VSMC phenotype switching is a core pathological process in vascular remodeling [6, 7]. More importantly, recent single-cell RNA-sequencing showed that VSMCs contribute to the pathophysiology of vascular remodeling via transdifferentiating into “inflammatory” macrophage-like cells, which results in increased vascular inflammation [8]. Furthermore, whole exome sequencing revealed that angiogenetic process imbalance elicited by dysregulated proliferation/apoptosis of endothelial cells and VSMCs might contribute to cerebrovascular malformation [9, 10]. These observations provide novel insights into cellular mechanisms underlying the alteration of vascular structure and function. Despite the accumulating evidence showed that phenotypic switching of VSMCs involves a complex regulatory network constituted by cytokines/growth factors [11], signal transduction molecules [12], transcription factors [13], non-coding RNA [14], epigenetic regulatory factors [15], etc., inflammation and oxidative stress are key mechanisms leading to VSMC phenotype switching and subsequent neointimal hyperplasia [2]. Thus, elucidation of molecular pathways that trigger inflammation and VSMC phenotype switching may provide helpful insights into the mechanisms underlying vascular remodeling.

Nucleotide-binding oligomerization domain-like receptor protein 3 (NLRP3) inflammasome activation potently stimulates inflammatory response via the release of the pro-inflammatory mediators interleukin-1β (IL) and IL-18 [16]. A recent study showed that monocyte promotes VSMC phenotypic switching through activating NLRP3 inflammasome, and increased IL-1β in phenotypically switching VSMCs is observed in the aortic roots of VSMC lineage tracing mice fed high cholesterol diet as well as in human atherosclerotic plaques [17]. This suggests that NLRP3 inflammasome activation might be implicated in vascular remodeling. As research on inflammasome progresses, it becomes clear that NLRP3 inflammasome is a cytoplasmic multiprotein complex consisting of the sensor NLRP3, apoptosis-associated speck-like protein (ASC), and caspase-1 [18, 19]. In response to harmful stimuli derived from pathogens, damaged or dead cells, and irritants, NLRP3 is activated and recruits ASC and pro-caspase-1, and the assembly of NLRP3, ASC, and pro-caspase-1 results in self-cleavage and activation of pro-caspase-1, leading to the secretion of bioactive IL-1β and IL-18 as well as the induction of pyroptosis via cleaving GSDMD into N-GSDMD and C-GSDMD [20, 21]. Nuclear factor-κB (NF-κB) is a key regulator of inflammation and plays a crucial role in neointimal hyperplasia [22]. Previous studies have demonstrated that NLRP3 expression in macrophages is regulated by NF-κB binding to its promoter [23]. Although important roles for NF-κB and NLRP3 inflammasome in vascular remodeling have been revealed, how NF-κB and NLRP3 are regulated during VSMC phenotype switching remains to be fully elucidated.

Recently, a large number of non-coding RNAs (ncRNAs) have emerged as important regulators in biological control and pathology [24]. Circular RNAs (circRNAs) are a class of non-coding RNAs with a covalently closed loop structure [25]. They can act as micro-RNA sponges, protein sponges, protein decoys, and scaffolds for the formation of protein complexes and are associated with the physiology and pathology of cardiovascular diseases [26–28]. circACTA2 is the first functional circRNA identified in VSMCs by our laboratory [29]. This circRNA is derived from circularization of the exon-5 to exon-9 of the smooth muscle α-actin gene and regulates VSMC differentiation and contractile function through interacting with miR-548f-5p. Additionally, we found that circACTA2 can modulate cellular senescence in human VSMCs by competing with CDK4 mRNA to bind to ILF3 and thus decreasing the ILF3 association with CDK4 mRNA, which reduces CDK4 mRNA stability [30]. These findings imply that circACTA2 might participate in multiple physiological and pathological processes in VSMCs*.* However, the roles of circACTA2 in the inflammation of VSMCs and the underlying mechanisms remain to be explored. In this study, we investigated whether and how circACTA2 modulates VSMC inflammation and neointimal hyperplasia by regulating NLRP3 expression and inflammasome activation.

## Materials and methods

### Human specimen collection

Human vascular specimens were obtained from 6 hypertensive patients and 4 non-hypertensive patients. Patients who had high blood pressure for more than 10 years used antihypertensive drugs to control their blood pressure. The renal arteries used in this study were sourced from the Second Hospital of Hebei Medical University (Shijiazhuang, China) between 2020 and 2022. The human study protocol was approved by the ethics committee of the Second Hospital of Hebei Medical University. All patients signed an informed consent form before donating tissue. A part of every renal artery was fixed overnight in 4% paraformaldehyde solution and conventional paraffin embedding was performed. Another portion of the renal arteries was placed in liquid nitrogen for rapid freezing and preserved at − 80 °C for nucleic acid extraction.

### Generation of transgenic mice overexpressing circACTA2 and animal experiments

The DNA fragments encoding circACTA2 were cloned into eukaryotic expression vector pβ-actin 2(+) and then transfected into VSMCs. qPCR assay showed that the expression of circACTA2 in VSMCs increased by 52 times than that of the empty vector-transfected cells. Next, pβ-actin 2(+)-circACTA2 was linearized with Sca I, and then, the linear vector was purified by Sephadex G-50. A final concentration of 5 ng/μl purified products was micro-injected into the pronuclei of fertilized oocytes of C57BL/6 × B6D2F1 mice, and then, the survived eggs were transplanted into pseudo-pregnant mice. The genotype of the newborn mice was identified by PCR with the genomic DNA extracted from tail tissues. All animal studies were approved by the Institutional Animal Care and Use Committee of Hebei Medical University and every effort was made to minimize suffering. 8–12 weeks old male C57BL/6 wild-type or circACTA2 transgenic mice were anesthetized with 1.5% isoflurane and the femoral artery was subjected to wire injury. 14 days later, all mice were anesthetized, serum was collected for ELISA analysis, and the femoral artery was harvested for RNA, and morphological and histological analysis.

### Cell culture and transfection

Mouse aortic smooth muscle cells (MASMCs) (ATCC, No. CRL-2797TM) were plated on 100-mm culture dishes at the density of 1 × 10^6^, and the experiments were initiated when the cells reached 75% confluence. MASMCs were routinely cultured in low-glucose Dulbecco’s modified Eagle’s medium (DMEM, Gibco Life Technologies, Rockville, MD) containing 100 units/ml penicillin, 100 μg/ml streptomycin, and 10% fetal bovine serum (GEMINI, USA) in a humidified incubator at 37 °C and 5% CO_2_.circACTA2 overexpression vector was constructed as follows: First, we constructed a circ-pcDNA3.1 vector with a reverse repeat sequence combined with 5′ donor splice sequences and 3′ acceptor splice sequences (Supplementary Table I). Between the donor splice site and acceptor splice site, we inserted a EcoNI enzyme site with sequences of 5′-CCTCAG∨CTAGG-3′ and a PmlI enzyme site with sequences of 5′-CAC ∨ GTG-3′. Then, the full-length sequence of circACTA2 was inserted to EcoNI and PmlI-digested circ-pcDNA3.1 with one-step cloning, as previously described [31]. pcDNA3.1 was used as a control. MASMCs were transfected with these vectors and incubated in serum-free medium for 24 h and then were stimulated with 25 ng/ml TNF-α for the indicated times (ProteinTech).

### Morphometry and histology

After euthanasia of mice, the femoral arteries were excised and immersed in 4% paraformaldehyde, dehydrated, and embedded in paraffin. The obtained human renal arteries were fixed overnight in 4% paraformaldehyde and subjected to the conventional gradient dehydration and paraffin embedding. Serially prepared sections (4 μm) were stained with hematoxylin and eosin (H&E). Images were acquired using a Leica microscope (Leica DM6000B, Switzerland) and digitized using LAS V.4.4 (Leica).

### Immunostaining

Immunofluorescence staining was performed using 4 μm paraffin slices of mouse femoral arteries and human renal arteries. After dewaxing and rehydration, the sections were preincubated with 10% normal goat serum for 30 min and then incubated with anti-TNF-α (60291-1-Ig, ProteinTech), anti-NLRP3 (19771-1-AP, ProteinTech), anti-Ki67 (27,309-1-AP, ProteinTech), anti-PCNA (10205-2-AP, ProteinTech), anti-GSDMD (20770-1-AP, ProteinTech), and anti-α-SMA (sc-53142, Santa Cruz) primary antibodies. Secondary antibodies were goat anti-rabbit 488 (ab150077, Abcam), goat anti-rabbit 647 (ab150079, Abcam), or goat anti-mouse 488 (ab150113, Abcam). Slices were sealed using a DAPI-containing sealer (H0621-V341, SouthernBiotech). Images were captured by a confocal microscope (DM6000 CFS, Leica) and processed by LAS X software. Immunohistochemical staining was performed using 4 μm paraffin slices of mouse femoral arteries. The sections were stained using anti-IL-1β (66737-1-Ig, ProteinTech), anti-IL-18 (10663-1-AP, ProteinTech), anti-IL-6 (21865-1-AP, ProteinTech), and anti-TNF-α (60291-1-Ig, ProteinTech). For immunofluorescence staining of mouse VSMCs, cultured cells on a slide were fixed with 4% paraformaldehyde for 15 min, then rinsed with PBS, and sealed with 10% normal goat serum for 30 min. Cells were incubated overnight at 4 °C with anti-NLRP3 (sc-134306, Santa Cruz), anti-Caspase-1 (22915-1-AP, ProteinTech), anti-ASC (10500-1-AP, ProteinTech), anti-p65 (sc-8008, Santa Cruz), anti-p50 (14220-1-AP, ProteinTech), and anti-GSDMD (20770-1-AP, ProteinTech), respectively. After PBS washing, fluorescein-labeled secondary antibodies were incubated for 1 h at room temperature and the slides were sealed with a DAPI-containing sealer. Confocal microscopy was performed using a Leica laser scanning confocal microscope (Leica DM6000B, Switzerland).

### Western blot analysis

Proteins from cells or tissues were prepared with RIPA (32010A, BBoxiProbe) lysis buffer. Equal amounts of proteins were separated by SDS-PAGE and electrotransferred to PVDF membranes (03010040001, Roche). PVDF membranes were incubated in TTBS containing 5% milk for 2 h at room temperature and incubated with primary antibody overnight at 4 °C. The following antibodies were used: anti-IL-18 (10663-1-AP, ProteinTech), anti-IL-1β (66737-1-Ig, ProteinTech), anti-TNF-α (60291-1-Ig, ProteinTech), anti-IL-6 (21865-1-AP, ProteinTech), anti-NLRP3 (19771-1-AP, ProteinTech), anti-Caspase-1 (22915-1-AP, ProteinTech), anti-ASC (10500-1-AP, ProteinTech), anti-IκB (10268-1-AP, ProteinTech), anti-p65 (sc-8008, Santa Cruz), anti-p50 (14220-1-AP, ProteinTech), anti-IKKα (ab32041, Abcam), anti-GSDMD (ab219800, Abcam), anti-GAPDH (10494-1-AP, ProteinTech), anti-Lamin B (66095-1-Ig, ProteinTech), and anti-β-actin (66009-1-Ig, ProteinTech). Enzyme-labeled secondary antibodies were incubated for 1 h at room temperature, and then, the band was detected with ECL (enhanced chemiluminescence) Fuazon Fx (Vilber Lourmat). Images were acquired and processed using FusionCapt Advance Fx5 software (Vilber Lourmat). All experiments were repeated three times.

### Isolation of RNA and PCR

Total RNA was extracted from cells or tissues according to the instructions of the Omega Total RNA Extraction Kit. The quality of the RNA was determined using a Nanodrop 2000 (Thermo). Reverse transcription was performed using MonScript™ RTIII All-in-One Mix (MR05401). qRT-PCR experiments for mRNA or circRNA using MonAmp™ ChemoHS qPCR Mix (MQ00401) were performed on an ABI 7500 FAST system (Life Technologies). The relative amounts of transcripts were normalized with 18SrRNA and calculated using the formula2^−ΔΔ^Ct.

### Immunoprecipitation assay

Magnetic beads (HY-K0202, MCE) were conjugated with anti-ASC, anti-NLRP3, or anti-p50 for 30 min at room temperature, and then washed four times using buffer. The bead-antibody mixture was then incubated with cell lysate for 30 min at room temperature, and the magnetic beads were washed four times using buffer. The bound proteins were separated using SDS-PAGE, followed by Western blot analysis with anti-NLRP3, anti-Caspase-1, anti-ASC, or anti-p65 antibodies.

### ELISA

The concentrations of IL-1β, IL-18, TNF-α, and IL-6 were measured in mouse serum, and IL-1β and IL-18 concentrations were determined in the culture medium of TNF-α-treated VSMCs overexpressing circACTA2. The absorbance was measured at 450 nm with a microplate reader (SPECTRAFluor Plus, Tecan) using an ELISA (ProteinTech) kit.

### RNA pull-down assay

The biotin-labeled RNA pull-down assay was performed as previously described [31]. Briefly, we cross-linked VSMCs in PBS with 1% formaldehyde for 10 min and then stopped the reaction with 0.125 M glycine. The cells were re-suspended with lysis buffers containing complete proteolytic enzyme inhibitors and ribonuclease inhibitors and then treated with ultrasound. 2X volume of hybridization buffer and 100 pmol of biotin-labeled probe were added to the cell lysates. Streptavidin microspheres (Life Technologies) were blocked with yeast trans-ribonucleic acid and bovine serum albumin for 2 h. 100 μl washed/blocked microspheres were added to every 100 pmol biotin-labeled probe and rotated at 37 °C for 30 min. The microspheres were captured with magnets (Life Technologies) and washed five times with wash buffer. The proteins of the microspheres were then extracted with elution buffer. After separation by SDS-PAGE, they were detected by Western blot analysis.

### RNA immunoprecipitation assay (RIP)

RIP was performed as previously described [31]. In brief, VSMCs were collected and lysed by NP40 lysis buffer. Immunoprecipitation kit (10007D, Thermo Fisher), anti-p65, anti-p50, anti-IκB, or IgG were used for immunoprecipitation assays according to the manufacturer’s instructions. After the pellets were washed three times with buffer, RNA was extracted with RNA purification kit according to the manufacturer’s instructions. The RNA fraction isolated by RIP was quantified by Nanodrop 2000 (Thermo) and detected by qRT-PCR.

### Cell fractionation

NE-Per Nuclear and Cytoplasmic Extraction Reagent (Thermo Fisher Science) was used to extract cytoplasmic and nuclear proteins from VSMCs after the appropriate treatments. An inhibitor of protein hydrolase was added to prevent degradation of the proteins. The extracted proteins were collected from each fraction and then analyzed by Western blotting.

### Small interfering RNA transfection

Small interfering RNA (siRNA) targeting mouse p50 (si-p50) and circACTA2 (si-circACTA2) was synthesized by GenePharma (Shanghai, China). Transfection was performed using Lipofectamine 2000 according to the manufacturer’s instructions. After transfection, VSMCs were treated with TNF-α. Cells were then collected for immunofluorescence staining or lysis for western blot and PCR.

### Fluorescence in situ hybridization

Cells cultured on coverslips were fixed with 4% paraformaldehyde. Paraffin slices (4 μm) of human renal arteries were prepared, dewaxed and rehydrated for hybridization. In situ hybridization was performed using specific probes for circACTA2 under the guidance of the miRCURY LNA™ MicroRNA ISH Optimization Kit (EXIQON). The slices were hybridized with the fluorescently labeled probes by incubation in hybridization buffer (Exiqon) at 55 °C in a heat block (LabNet), and then washed with SSC buffer, and the nuclei were stained with DAPI (157,574, MB Biomedical). Images were acquired using a confocal laser microscope and digitized using LAS X (Leica) software.

### Protein purification

293 T cells transferred with Flag-tagged expression plasmids for p65, p50, or IκB were lysed with RIPA lysis buffer. Magnetic beads (HY-K0207) were washed two times with washing buffer and then incubated with cell lysate overnight at 4 °C. After the magnetic beads were washed three times using buffer, the bound proteins were eluted by adding acidic eluent.

### 3D Matrigel drop invasion assay

As previously described [32], 5 × 10^4^ VSMCs infected with lentivirus overexpressing circACTA2 were suspended in 10 μl of matrix gel and dropped as droplets into 24-well plates for 20 min to form matrix droplets, and then, DMEM medium-containing 2% fetal bovine serum was added and changed every 3 days, during which TNF-α was added to the cells for 6 days. The radial distance of cells migrating from the edge was measured as radial migration on the sixth day.

### Propidium iodide (PI) staining

Propidium iodide (PI) (HY-D0815) assay was used to assess the extent of cell apoptosis. circACTA2-overexpressed VSMCs were treated with TNF-α, washed with PBS and fixed with 4% paraformaldehyde. After staining with PI for 30 min, cells were washed with SSC and then observed with a fluorescence microscope.

### Scratch wound-healing assay

VSMCs were cultured in 6-well plates and transfected with circACTA2-expressing plasmids for 24 h, and then treated with TNF-α. Sterile pipette tips were used to scratch a wound on the surface of the confluent cell monolayer. 24 h later, images were taken under a light microscope.

### Statistical analysis of experimental data

All data are expressed as mean ± SEM. ANOVA and Student's t test were used to assess differences between the two groups. Multiple comparisons or repeated measures were analyzed using ANOVA or repeated ANOVA followed by Tukey’s post hoc test. When a value of *P* < 0.05 was present, we considered it statistically significant. Graphpad Prism 8 software (GraphPad Software, San Diego, CA, USA) was used to perform statistical analysis.

## Results

### circACTA2 is down-regulated in human intimal hyperplasia and its overexpression inhibits neointimal formation in mouse model of intimal hyperplasia

**Fig. 1:**
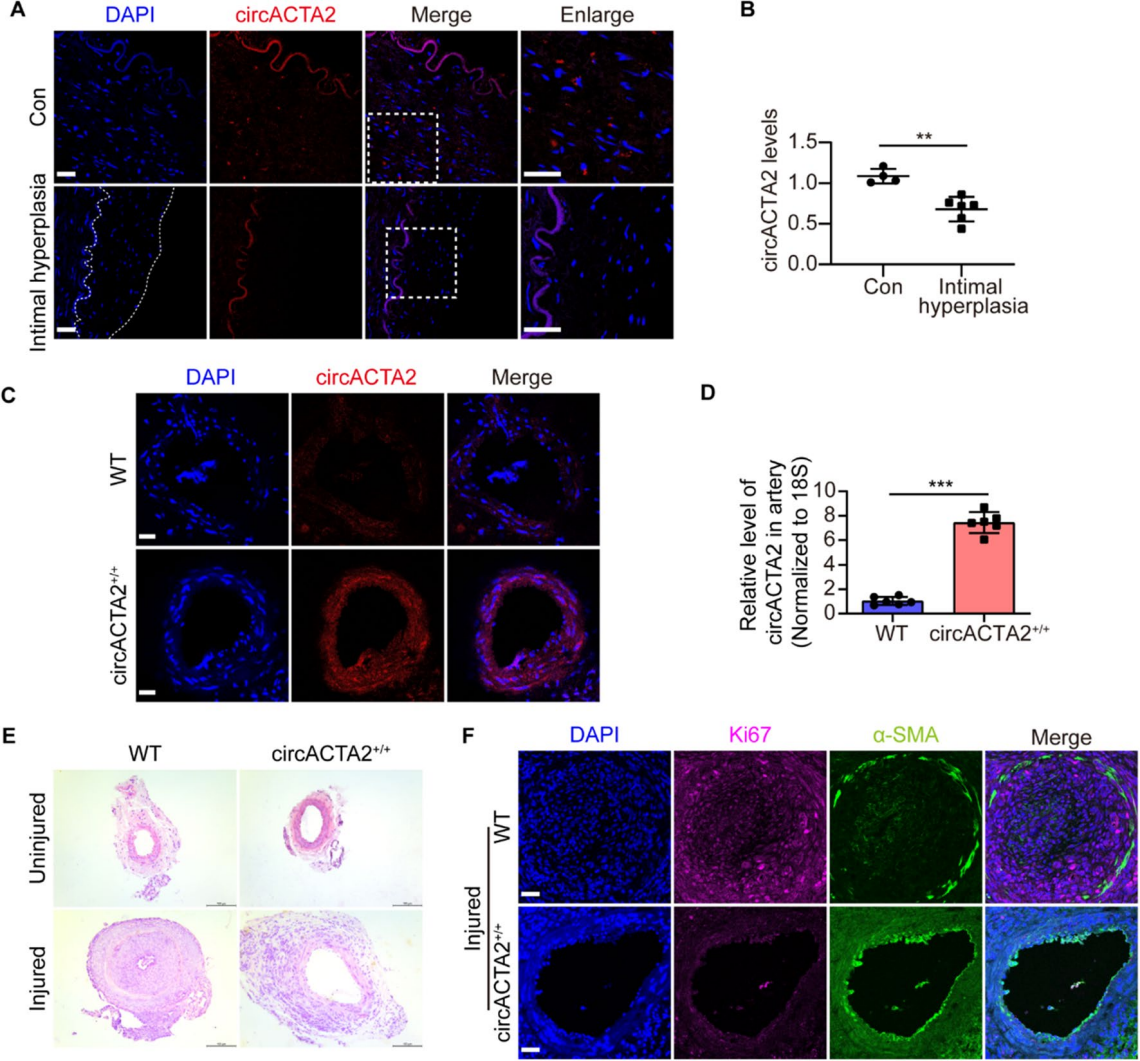
circACTA2 is down-regulated in human intimal hyperplasia and its overexpression inhibits neointimal formation in mouse model of intimal hyperplasia. **A** Expression of circACTA2 was detected by RNA in situ hybridization in the renal artery intimal hyperplasia of hypertensive patients, and neointimal hyperplasia was outlined by the dot line. Scale bars: 30 μm. **B** circACTA2 expression in the renal artery intimal hyperplasia of hypertensive patients was detected by qRT-PCR; **P < 0.01 vs. Con. n = 4 in Con group, *n* = 6 in the patient group. **C** Expression of circACTA2 was detected by RNA in situ hybridization in artery of WT and circACTA2^+/+^ mice. Scale bars: 20 μm. **D** Expression of circACTA2 in artery of WT and circACTA2^+/+^ mice was detected by qRT-PCR; ***P < 0.001 vs. WT. *n* = 6 per group. **E** Representative hematoxylin and eosin (HE)-stained cross-sections from uninjured and wire-injured arteries of WT and circACTA2^+/+^ mice. Scale bars: 100 μm. **F** Co-immunofluorescence staining for Ki67 (red), α-SMA (green), and DAPI (blue) in injured arteries of WT and circACTA2^+/+^ mice. Scale bars: 30 μm. Data are represented as mean ± SEM

To assess the role of circACTA2 in vascular remodeling, we first examined circACTA2 expression in the renal artery intimal hyperplasia (Fig. S1A) of hypertensive patients. RNA in situ hybridization and qRT-PCR analysis revealed that circACTA2 had a lower expression in human intimal hyperplasia than in control renal arteries (Figs. 1A, B and S1B, C). We then examined the effect of circACTA2 overexpression on neointimal formation. To do this, we generated circACTA2 transgenic mice (circACTA2^+/+^ mice). The genotype of transgenic mice was identified by PCR (Fig. S1D) and the expression level of circACTA2 in the artery wall was significantly higher in transgenic mice than in non-transgenic mice, as evidenced by qRT-PCR and FISH (fluorescence in situ hybridization) (Figs. 1C, D and S1E, F). More importantly, following femoral artery wire injury for 14 days, the neointima formation was significantly attenuated in the circACTA2^+/+^ mice relative to the wild-type (WT) controls, without difference in neointima thickness between uninjured circACTA2^+/+^ and wild-type mice, as confirmed by H&E staining (Figs. 1E and S1G). In parallel to the changes in neointimal hyperplasia, immunofluorescence staining of Ki67 and PCNA showed that overexpression of circACTA2 substantially reduced cell proliferation in the injured femoral artery compared with that of wild-type mice (Figs. 1F and S1H-J). These results suggested that circACTA2 is a necessary regulator of vascular remodeling.

### circACTA2 attenuates the neointima formation by inhibiting inflammation

**Fig. 2:**
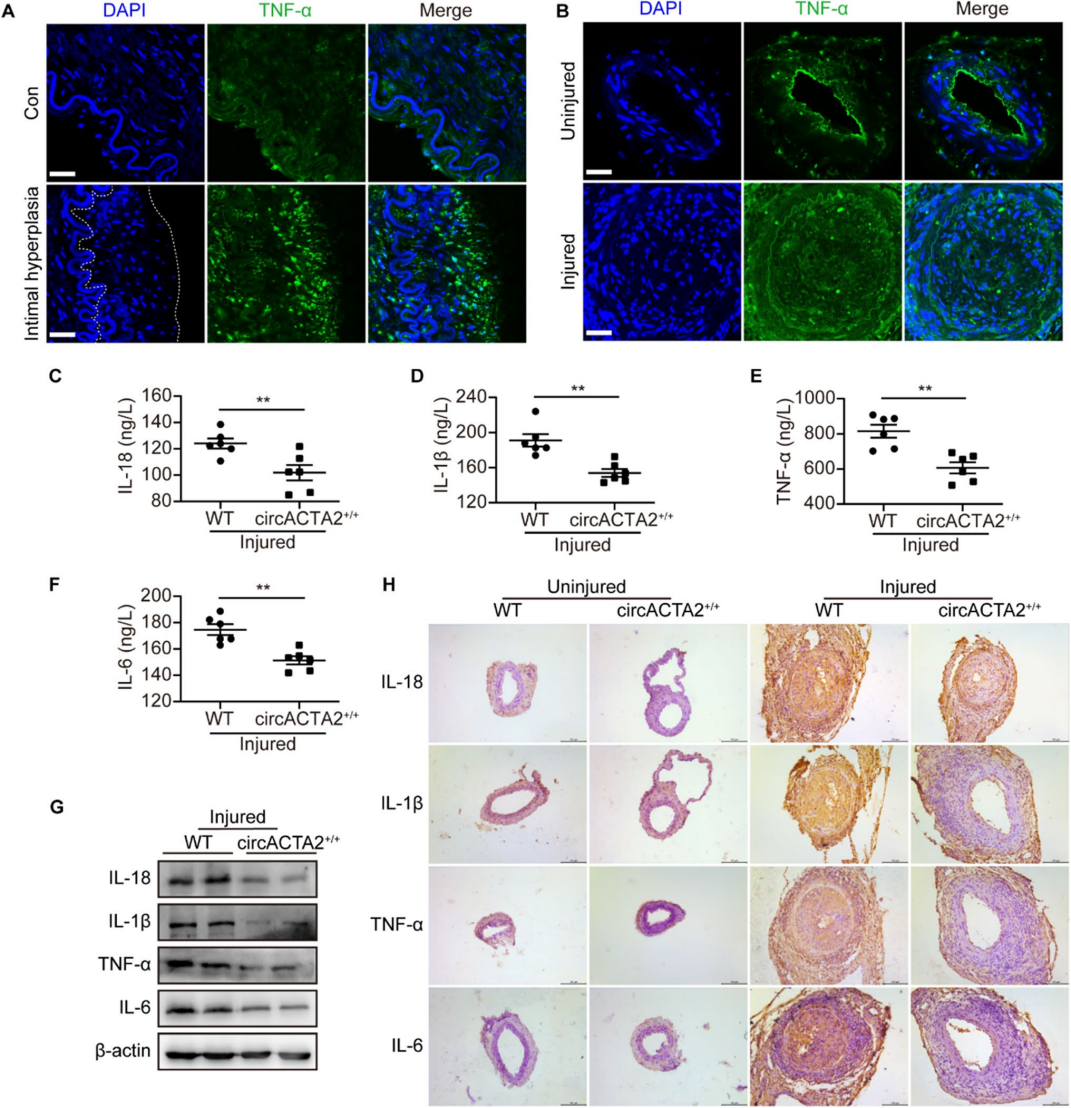
circACTA2 attenuates the neointima formation by inhibiting inflammation. **A** Immunofluorescence staining of TNF-α (green) and DAPI (blue) in the renal artery intimal hyperplasia of hypertensive patients, and neointimal hyperplasia was outlined by the dot line. Scale bars: 30 μm. **B** Immunofluorescence staining of TNF-α (green) and DAPI (blue) in uninjured and wire-injured arteries of WT mice. Scale bars: 30 μm. **C–F** IL-18 (**C**), IL-1β (**D**), TNF-α (**E**), and IL-6 (**F**) content in serum of WT and circACTA2^+/+^ mice was determined by ELISA. **P < 0.01 vs. WT. *n* = 6 per group. **G** The expression of IL-18, IL-1β, TNF-α, and IL-6 in injured arteries of WT and circACTA2^+/+^ mice was detected by Western blotting. **H** Immunohistochemical staining of cross-sections detected the expression of IL-18, IL-1β, TNF-α, and IL-6 in uninjured and wire-injured arteries of WT and circACTA2^+/+^ mice. Scale bars: 100 μm. Data are represented as mean ± SEM

Inflammation plays a critical role in the vascular response to injury, which results in the proliferation of VSMCs and extracellular matrix protein production and eventually leads to vascular remodeling [33]. Therefore, we wondered whether circACTA2-suppressed neointimal formation was of relevance to its inhibition of vascular inflammation. Consistent with our previous results [34], the expression of TNF-α was significantly elevated in human intimal hyperplasia of renal arteries as well as in mouse intimal hyperplasia of femoral arteries at 14 days after injury (Figs. 2A, B and S2A-D). Subsequently, we examined the inflammatory factors in the serum of mice subjected to wire injury of the femoral artery. ELISA assay showed that the expression levels of IL-18, IL-1β, TNF-α, and IL-6 were significantly lower in circACTA2^+/+^ mice than in wild-type mice (Fig. 2C–F). Also, the expression levels of these inflammatory factors in the injured femoral arteries of circACTA2^+/+^ mice were obviously reduced compared to wild-type mice, as shown by western blotting (Fig. 2G). A similar result was obtained by immunohistochemistry staining, with circACTA2 overexpression reducing neointimal hyperplasia induced by wire injury (Fig. 2H). Together, these findings suggest that circACTA2 attenuates the neointima formation by inhibiting inflammation.

### circACTA2 alleviates VSMC inflammation by suppressing the activation of NLRP3 inflammasome

**Fig. 3:**
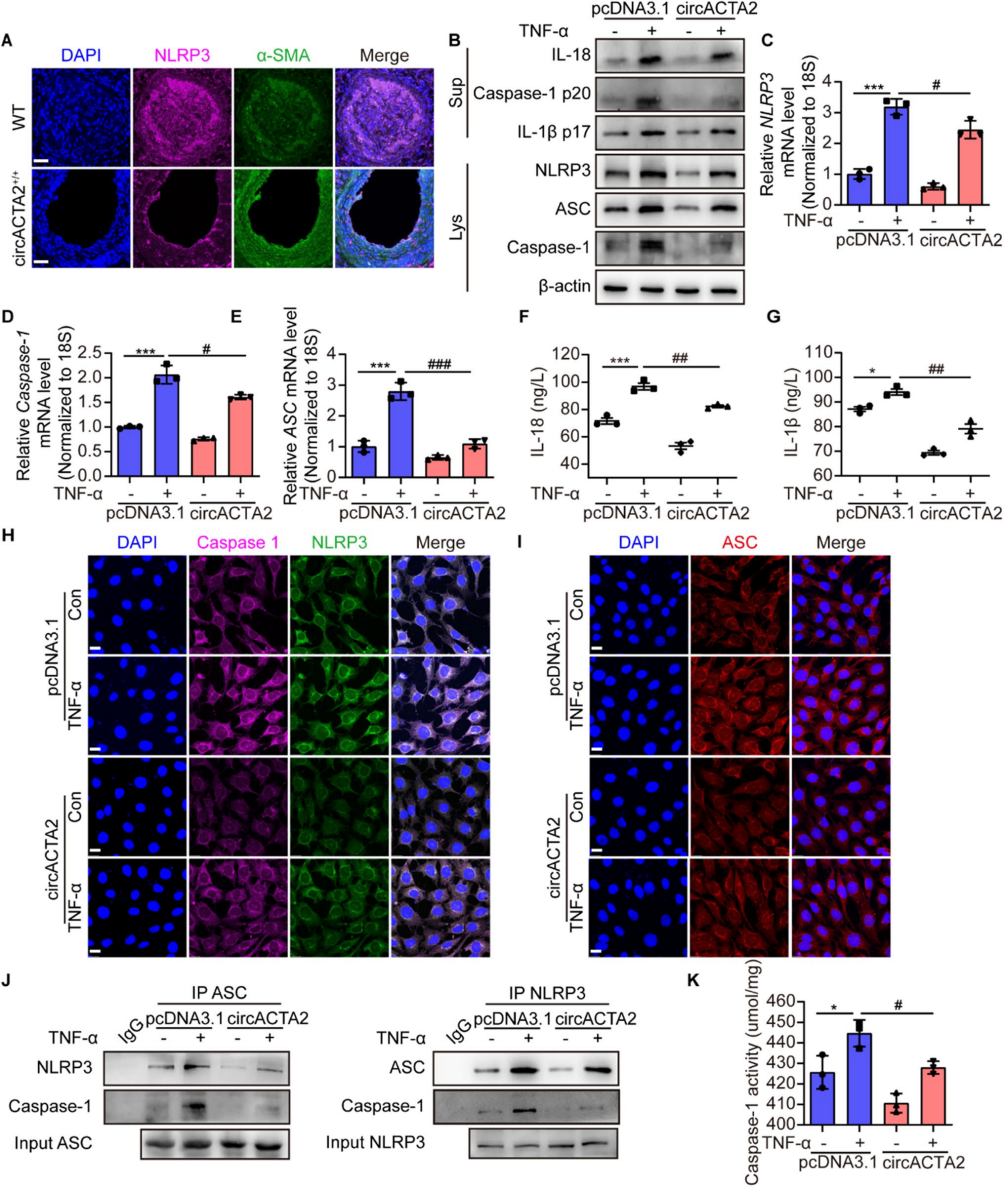
circACTA2 alleviates VSMC inflammation by suppressing the activation of NLRP3 inflammasome. **A** Co-immunofluorescence staining for NLRP3 (red), α-SMA (green), and DAPI (blue) in injured arteries of WT and circACTA2^+/+^ mice. Scale bars: 30 μm. **B** Western blotting detected the expression of IL-18, Caspase-1 (p20), IL-1β (p17), NLRP3, Caspase-1, and ASC in the supernatant and cellular lysates of circACTA2-overexpressed VSMCs, which were stimulated with or without TNF-α (25 ng/ml) for 24 h. **C–E** NLRP3 (**C**), Caspase-1 (**D**), and ASC (**E**) mRNA expression was detected by qRT-PCR in VSMCs treated as in (**B**). ***P < 0.001 vs. control; ^#^*P* < 0.05, ^###^*P* < 0.001 vs. pcDNA3.1. *n* = 3 per group. (**F**, **G**) IL-18 (**F**) and IL-1β (**G**) content determined by ELISA in the supernatant of VSMCs treated as in (**B**). *P < 0.05, ***P < 0.001 vs. Control; ^##^*P* < 0.01 vs. pcDNA3.1. *n* = 3 per group. **H** Co-immunofluorescence staining for Caspase-1 (red), NLRP3 (green), and DAPI (blue) in VSMCs treated as in (**B**). Scale bars: 20 μm. **I** Immunofluorescence staining for ASC (red) and DAPI (blue) in VSMCs treated as in (**B**). Scale bars: 20 μm. **J** CoIP analysis of interaction between NLRP3, Caspase-1, and ASC in cellular lysates of VSMCs treated as in (**B**). **K** Caspase-1 enzymatic activity assay in VSMCs treated as in (**B**). *P < 0.05 vs. control; ^#^*P* < 0.05 vs. pcDNA3.1. *n* = 3 per group. Data are represented as mean ± SEM

Since secretion of IL-1β and IL-18 requires the activation of NLRP3 inflammasome [35] and the latter is known to be an important mechanism driving VSMC proliferation [17], we explored whether NLRP3 expression is upregulated in VSMCs in neointimal hyperplasia induced by wire injury*.* We used immunofluorescence staining to examine VSMCs and found that NLRP3 was primarily colocalized with VSMC marker SM α-actin, indicating that VSMCs in the neointimal hyperplasia express NLRP3 (Figs. 3A and S3A, B)*.* To further clarify whether circACTA2 suppresses VSMC inflammation through inhibiting NLRP3 inflammasome activation, we overexpressed circACTA2 in mouse VSMCs by transfecting circACTA2 expression plasmids (Fig. S3C) and then treated cells with TNF-α and detected the expression of NLRP3 inflammasome components NLRP3, ASC, and procaspase-1 as well as IL-1β and IL-18*.* The results showed that TNF-α, an activator of NLRP3 inflammasome, significantly stimulated the mRNA and protein expression of NLRP3, ASC, and caspase-1 in empty vector-transfected VSMCs, which was markedly alleviated by overexpression of circACTA2 in VSMCs (Fig. 3B–E). Accordingly, TNF-α treatment significantly increased the mature (cleaved) forms of IL-1β and IL-18 in cell culture supernatants*,* while their level was reduced in circACTA2-overexpressed VSMCs, as shown by Western blot analysis (Fig. 3B) and ELISA assay (Fig. 3F, G). To further validate the inhibitory effect of circACTA2 on NLRP3 inflammasome, we knocked down the expression of circACTA2 in VSMCs with the specific siRNA targeting circACTA2 and examined the effect of TNF-α on NLRP3 inflammasome activation. In contrast to overexpression of circACTA2, treating circACTA2-silencing VSMCs with TNF-α resulted in the further upregulation of the mRNA and protein levels of NLRP3, ASC, and caspase-1. ELISA assay of IL-1β and IL-18 showed the same results (Fig. S3E–J).

Further, confocal immunofluorescence staining of NLRP3 and caspase-1 showed that treatment of VSMCs with TNF-α markedly enhanced NLRP3 and caspase-1 expression, with an obvious increase in NLRP3 co-localization with caspase-1, while these effects were dramatically inhibited by circACTA2 overexpression in VSMCs (Figs. 3H and S3K, M). Simultaneously, TNF-α significantly increased the expression level of ASC in empty vector-transfected VSMCs, which was largely abrogated by circACTA2 overexpression (Figs. 3I and S3L, N). Further, we examined the effects of TNF-α or/and circACTA2 overexpression on the formation of inflammasome complex and confirmed that TNF-α markedly increased the interaction among the ASC, NLRP3, and caspase-1, whereas circACTA2 overexpression could effectively abrogate the formation of inflammasome complex, as shown by co*-*immunoprecipitation analysis. Likewise, a similar result was obtained by reciprocal immunoprecipitation with anti-NLRP3 antibodies, showing that the interaction among the ASC, NLRP3, and caspase-1 could be obviously repressed by circACTA2 overexpression (Fig. 3J). Correspondingly, the increase of caspase-1 enzyme activity induced by TNF-α was blunted by overexpressing circACTA2 in VSMCs (Fig. 3K). Taken together, these findings clearly suggested that circACTA2 inhibits VSMC inflammation by suppressing the activation of NLRP3 inflammasome.

### circACTA2 inhibits the expression of NF-κB p65 and p50 subunits and interacts with them

**Fig. 4:**
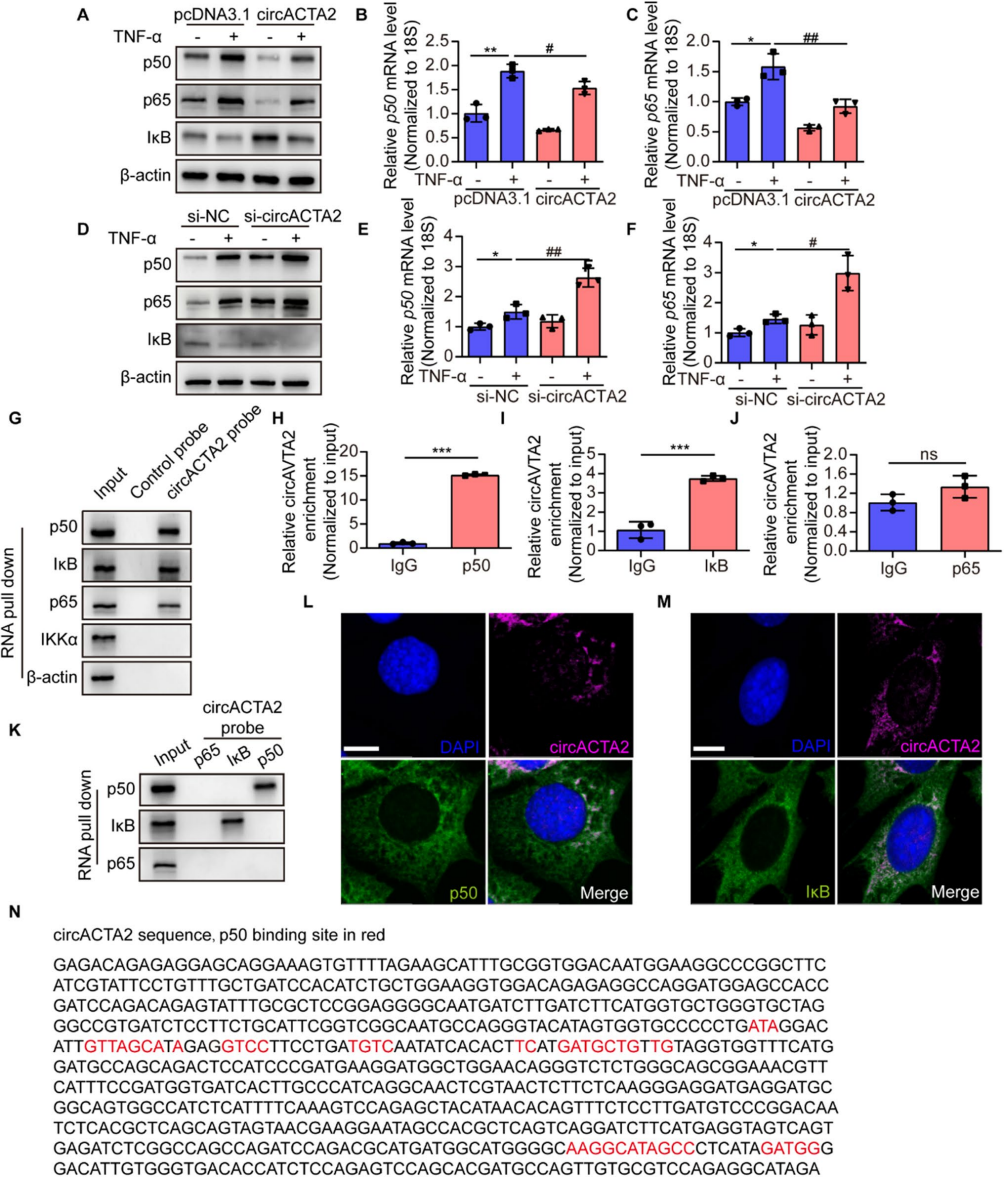
circACTA2 inhibits the expression of NF-κB p65 and p50 subunits and interacts with them. **A** Western blot analysis detected the expression of p65, p50, and IκB in circACTA2-overexpressed VSMCs, which were stimulated with or without TNF-α (25 ng/ml) for 24 h. **B**,** C** p50 (**B**) and p65 (**C**) mRNA expression detected by qRT-PCR in VSMCs treated as in (**A**). *P < 0.05, **P < 0.01 vs. Control; ^#^*P* < 0.05, ^##^*P* < 0.01 vs. pcDNA3.1. *n* = 3 per group. **D** Western blot analysis detected the expression of p65, p50, and IκB in circACTA2-knocked down VSMCs, which were stimulated with or without TNF-α (25 ng/ml) for 24 h. **E**, **F** p50 (**E**) and p65 (**F**) mRNA expression detected by qRT-PCR in VSMCs treated as in (**D**). *P < 0.05 vs. Control; ^#^*P* < 0.05, ^##^*P* < 0.01 vs. pcDNA3.1. *n* = 3 per group. **G** VSMC lysates were pulled down with circACTA2 probe and detected by Western blotting with anti-p50, anti-IκB, anti-p65, and anti-IKKα antibodies. **H–J** qRT-PCR detected circACTA2 levels in the RNA–protein immunoprecipitates pulled down by anti-p50 (**H**), anti-IκB (**I**), and anti-p65 (**J**) antibodies in VSMCs. ***P < 0.001 vs. IgG. *n* = 3 per group. **K** RNA pull-down assay was performed to determine the interaction between circACTA2 and purified p50, IκB, and p65. **L**, **M** Co-immunofluorescence staining for circACTA2 (red), p50 (green), IκB (green), and DAPI (blue) in VSMCs. Scale bars: 10 μm. **N** The interaction sites between circACTA2 and p50 were predicted by HDOCK website (http://hdock.phys.hust.edu.cn/). Data are represented as mean ± SEM

NF-κB-mediated signaling is well known to regulate NLRP3 and pro-IL-1β expression [21, 36]. To explore the underlying mechanism(s) by which circACTA2 suppresses NLRP3 inflammasome activation, we focused on the effects of circACTA2 on NF-κB signaling in the following study. We found that overexpression of circACTA2 in VSMCs significantly suppressed the promoting effect of TNF-α on the mRNA and protein expression of p65 and p50 subunits, while upregulating the expression of the inhibitory subunit IκB (Fig. 4A–C). In contrast, silencing circACTA2 resulted in the further upregulation of the p65 and p50 induced by TNF-α, which was accompanied with downregulation of the expression of IκB (Fig. 4D–F). Considering that an important function of circRNAs is able to interact with proteins to regulate their subcellular localization and functions [37], we examined whether circACTA2 interacts with NF-κB. RNA pull-down followed by immunoblotting showed that circACTA2 interacted with all the three subunits (p65, p50, and IκB) of NF-κB (Fig. 4G). Further, we used RNA immunoprecipitation (RIP) performed with anti-p65, anti-p50, or anti-IκB to validate their interaction. qRT-PCR showed that among these three subunits, p50 had a stronger affinity to circACTA2 than the IκB (Fig. 4H, I), but the affinity of p65 for circACTA2 was not significantly different from that of IgG (Fig. 4J). These results imply that circACTA2 specifically interacts with p50. To further test whether circACTA2 directly associates with p65, p50, and IκB, we constructed their expression plasmids, tagged with Flag tags, and overexpressed them in 293 T cells (Fig. S4A). After purification of the p65, p50, and IκB proteins, their binding with circACTA2 was detected by RNA pull down followed by immunoblotting. The results revealed a direct interaction between circACTA2 and p50 or IκB, but not p65 (Fig. 4K). Moreover, there was a marked co-localization between circACTA2 and p50 or IκB, as detected by immunofluorescence staining for circACTA2 probe and p50 or IκB antibody (Fig. 4L, M and S4B-D). Indeed*,* there exist the potential binding sites between circACTA2 and p50 or IκB, as predicted by bioinformatics analysis by HDOCK website (http://hdock.phys.hust.edu.cn/) (Figs. 4N and S4E). Taken together, these results suggest that circACTA2 exerts its inhibitory effect on inflammasome activation by interacting with p50 and IκB.

### circACTA2 impedes the p65 nuclear translocation induced by TNF-α via interacting with p50

**Fig. 5:**
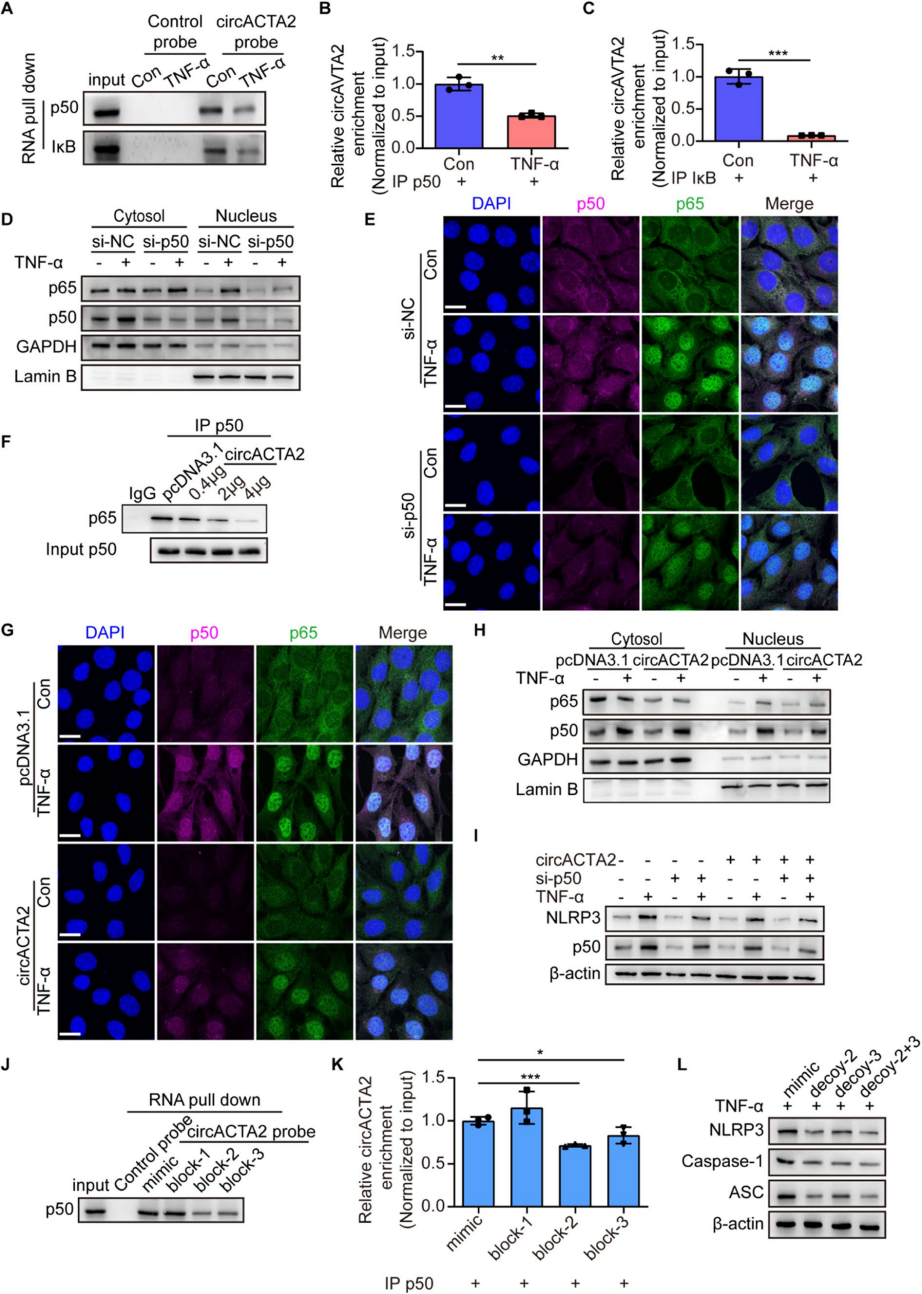
circACTA2 impedes the p65 nuclear translocation induced by TNF-α via interacting with p50. **A** VSMCs were treated with or without TNF-α (25 ng/ml) for 24 h, and then, cellular lysates were pulled down with circACTA2 probe and detected by Western blotting with anti-p50 and anti-IκB antibodies. **B**, **C** qRT-PCR detected circACTA2 levels in the RNA–protein immunoprecipitates pulled down by anti-p50 (**B**) and anti-IκB (**C**) antibodies in VSMCs treated as in (**A**). **P < 0.01, ***P < 0.001 vs. Con. *n* = 3 per group. **D** Western blot analysis detected the expression of p65 and p50 in the cytoplasmic and nuclear fractions of VSMCs transfected with si-p50 and then stimulated with or without TNF-α for 30 min. **E** Co-immunofluorescence staining for p50 (red), p65 (green), and DAPI (blue) in VSMCs treated as in (**D**). Scale bars: 20 μm. **F** CoIP analysis of interaction between p65 and p50 in VSMCs transfected with the different concentrations of circACTA2-expressing plasmid. **G** Co-immunofluorescence staining for p50 (red), p65 (green), and DAPI (blue) in VSMCs transfected with circACTA2-expressing plasmid and then stimulated with or without TNF-α for 30 min. Scale bars: 20 μm. **H** Western blot analysis detected the expression of p65 and p50 in cytoplasmic and nuclear fractions of VSMCs treated as in (**G**). **I** Western blot analysis detected the expression of NLRP3 and p50 in p50-knocked down or/and circACTA2-overexpressed VSMCs, which were stimulated with or without TNF-α (25 ng/ml) for 24 h. **J** RNA pull-down assay was performed to examine the interaction between circACTA2 and p50 in VSMCs transfected with different blocking oligos. **K** qRT-PCR detected circACTA2 levels in the RNA–protein immunoprecipitates pulled down by anti-p50 antibody in VSMCs transfected with different blocking oligos. *P < 0.05, ***P < 0.001 vs. mimic. *n* = 3 per group. **L** Western blot analysis detected the expression of NLRP3, Caspase-1, and ASC in VSMCs transfected with decoy oligos and stimulated with TNF-α (25 ng/ml) for 24 h. Data are represented as mean ± SEM

Given the important role of circACTA2 interaction with p50 and IκB in regulating the NF-κB signaling, we sought to determine how the NF-κB signaling is regulated by the interaction of circACTA2 with p50 or IκB. We first investigated whether treatment of VSMCs with TNF-α can affect the interaction between circACTA2 and p50 or IκB. As shown in Fig. 5A, the association of the control probe targeting vector with p50 or IκB was hardly detectable regardless of treatment with TNF-α, but TNF-α stimulation significantly attenuated the interaction between circACTA2 probe and p50 or IκB, as evidenced by RNA pull-down followed by immunoblotting. Correspondingly, the results of the RIP followed by qRT-PCR were similar to those seen in RNA pull-down assay, showing a significant decrease in circACTA2 binding to p50 or IκB after TNF-α treatment (Fig. 5B, C). Based on the above findings that circACTA2 could upregulate the protein level of IκB, an inhibitory subunit of NF-κB signaling, and interacted with IκB, we speculated that circACTA2 could exert its anti-inflammatory effect by binding to IκB to inhibit its degradation. To verify this, we overexpressed circACTA2 in VSMCs and then treated the cells with CHX for different times, and detected IκB expression. The results showed that overexpression of circACTA2 did not affect the half-life of IκB, without obvious increase in IκB stability (Fig. S5A).

It has been known that NF-κB complexes (p50/p65) are present in an inactive form in the cytoplasm, bound to IκB. Under certain stimuli, IκB is phosphorylated and degraded, enabling translocation of p50/p65 heterodimer into the nucleus to activate target gene transcription [38]. Therefore, we sought to know whether p50 interaction with circACTA2 affects its association with p65 and thus impedes p65 translocation into the nucleus. We first examined the effects of siRNA-mediated p50 knockdown on the subcellular distribution of p65. Immunoblotting and immunofluorescence staining of p65 and p50 showed that silencing p50 in VSMCs significantly reduced the nuclear distribution of p65 regardless of treatment with TNF-α (Figs. 5D, E and S5B–D). Further, we investigated the effect of circACTA2 overexpression in VSMCs on the interaction of p65 with p50. When increasing amounts of circACTA2 expression plasmids were applied to transfect VSMCs, the association of p65 with p50 progressively decreased as amounts of circACTA2 expression plasmids increased (Figs. 5F and S5E). Next, we examined the effect of circACTA2 overexpression on the subcellular localization of p65 and p50. The results of immunofluorescence staining revealed that overexpression of circACTA2 in VSMCs substantially suppressed the nuclear translocation of p65 and p50 induced by TNF-α (Figs. 5G and S5F, G). Western blot analysis also indicated that the expression level of p65 and p50 in the nucleus of circACTA2-overexpressed VSMCs was significantly lower than that of the control (Fig. 5H). To provide further evidence supporting the above findings, we co-transfected VSMCs with si-p50 and circACTA2 expression plasmids either alone or together and then treated the cells with TNF-α. The results demonstrated that the inhibitory effect of circACTA2 overexpression on p50 and NLRP3 expression was further strengthened in p50-silenced VSMCs, and the expression level of NLRP3 mRNA showed the same results as those of immunoblotting analysis (Figs. 5I and S5H). Besides, we blocked the binding of circACTA2 to p50 by transfecting VSMCs with blocking oligonucleotide, which is complimentary to the p50-binding site in the circACTA2 sequence. RNA pull-down and RIP assays revealed that p50 binding to circACTA2 was significantly reduced after the transfection of the blocking oligonucleotides block-2 and block-3 (Fig. 5J, K). Correspondingly, the expression of NLRP3 was moderately upregulated after blocking the binding of circACTA2 to p50 (Fig. S5I). Also, we employed a decoy oligonucleotide, which has a similar sequence to the p50-binding site in the circACTA2 sequence, to compete with circACTA2 for binding to p50, thus suppressing the binding between p50 and p65. The results showed that the transfection of the decoy oligonucleotides (decoy-2, decoy-3, and decoy-2 + 3) greatly attenuated the expression level of NLRP3, ASC, and caspase-1 (Fig. 5L). Overall, our results clearly indicated that circACTA2 suppresses the p65 nuclear translocation via interacting with p50 and thus inhibits inflammation in VSMCs.

### circACTA2 alleviates inflammation through repressing NLRP3 inflammasome activation-mediated VSMC pyroptosis

**Fig. 6:**
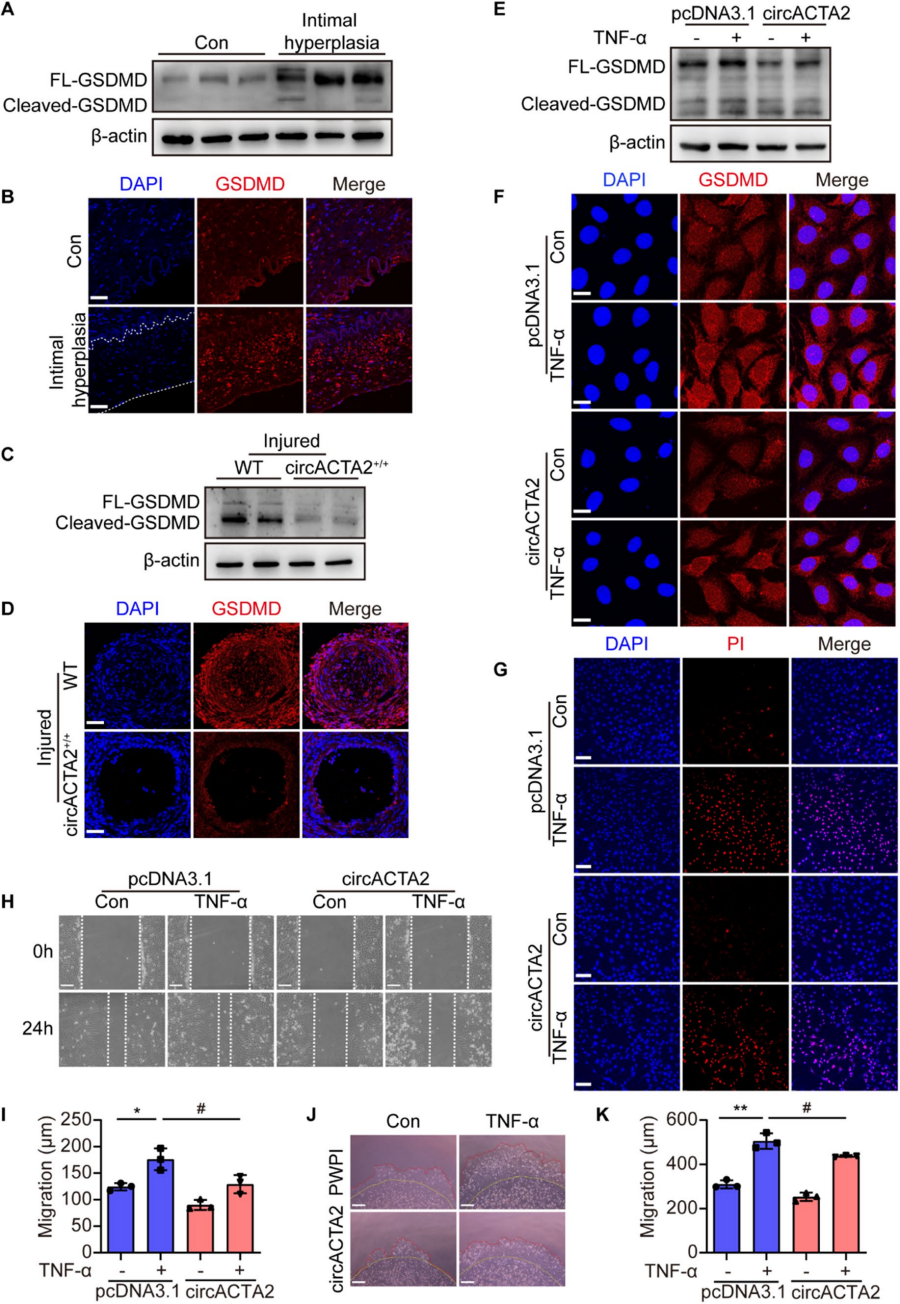
circACTA2 alleviates inflammation through repressing NLRP3 inflammasome activation-mediated VSMC pyroptosis. **A**, **B** Western blot analysis (**A**) and immunofluorescence staining (**B**) detected the expression of GSDMD in the renal artery intimal hyperplasia of hypertensive patients. And neointimal hyperplasia was outlined by the dot line. Scale bars: 40 μm. **C**,** D** Western blotting (**C**) and immunofluorescence staining (**D**) detected the expression of GSDMD in the injured arteries of WT and circACTA2^+/+^ mice. Scale bars: 40 μm. **E**,** F** Western blotting (**E**) and immunofluorescence staining (**F**) detected the expression of GSDMD in circACTA2-overexpressed VSMCs stimulated with or without TNF-α (25 ng/ml) for 24 h. Scale bars: 15 μm. **G** Propidium iodide (PI) staining assay of VSMCs treated as in (**E**,** F**). Scale bars: 80 μm. **H** Migratory ability assessed by wound-healing assay in VSMCs treated as in (**E**,** F**). Scale bars: 100 μm. **I** Migratory distance of VSMCs treated as in (**E**,** F**). *P < 0.05 vs. control; ^#^*P* < 0.05 vs. pcDNA3.1. *n* = 3 per group. **J** Matrigel invasion assay of circACTA2-overexpressed VSMCs treated with or without TNF-α (25 ng/ml). Scale bars: 250 μm. **K** Migratory distance of VSMCs treated as in (**J**). **P < 0.01 vs. control; ^#^*P* < 0.05 vs. PWPI. *n* = 3 per group. Data are represented as mean ± SEM

**Fig. 7:**
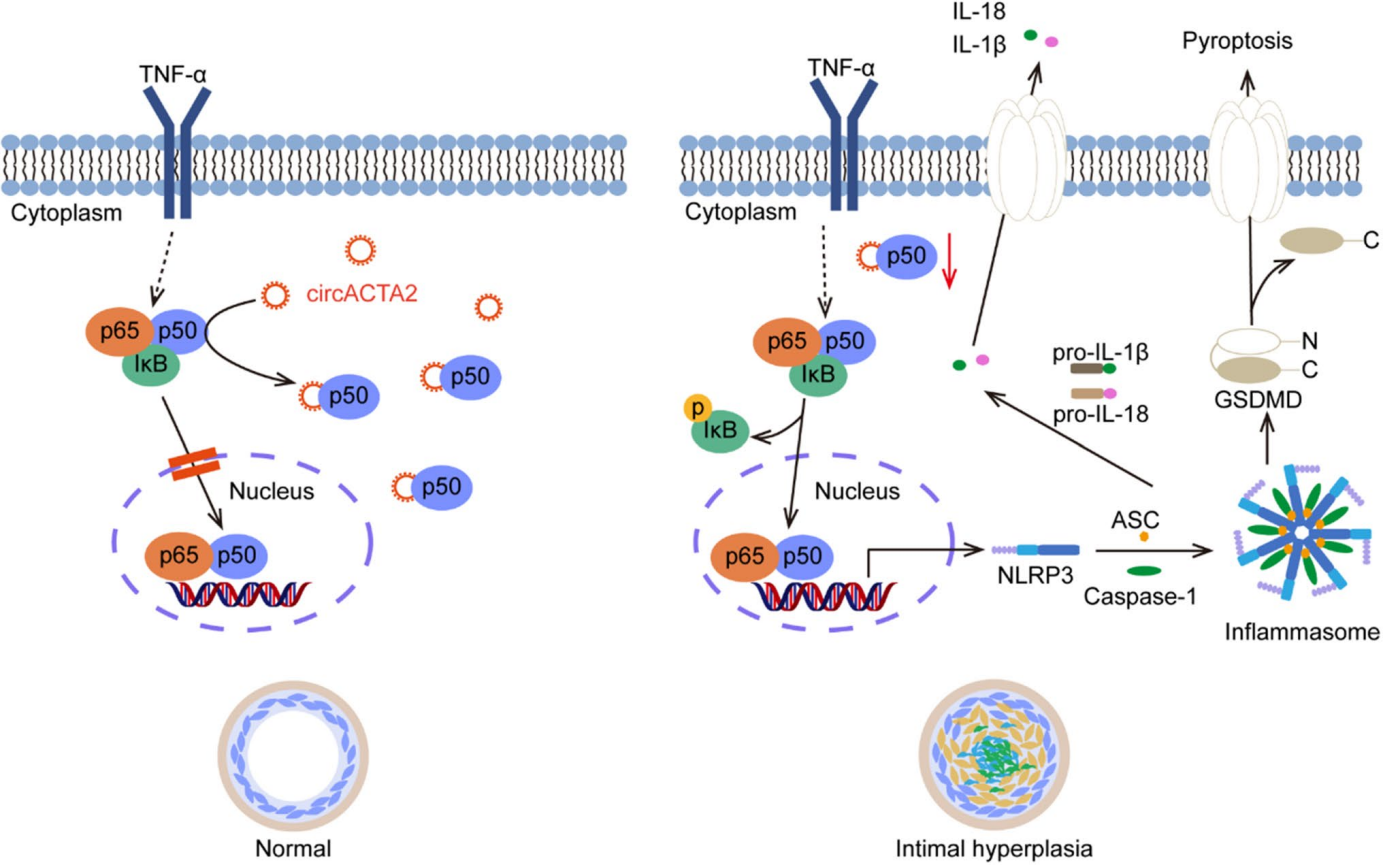
Schematic illustration of the mechanism whereby circACTA2 regulates NLRP3 inflammasome activation via interacting with p50. Under physiological conditions, circACTA2 interacts with NF-κB p50 in the cytoplasm, impeding the formation of the p50/p65 heterodimer and nuclear translocation, and thus inhibits NLRP3 inflammasome expression and activation. When circACTA2 expression is down-regulated in VSMCs, the interaction of circACTA2 with p50 is attenuated, which in turn facilitates the formation of the p50/p65 heterodimer and nuclear translocation, leading to vascular inflammation and remodeling

Because the activation of inflammasomes promotes the secretion of IL-1β and IL-18 and triggers pyroptosis, and the cleavage of gasdermin D (GSDMD), a crucial component of inflammasomes, by caspase-1 is crucial for pyroptosis and IL-1β secretion [39], we hypothesized that a relationship may exist between circACTA2 expression and NLRP3 inflammasome-mediated pyroptosis in VSMCs*.* To test this hypothesis, we first detected GSDMD expression and cleavage in human intimal hyperplasia of renal arteries. Immunoblotting analysis and immunofluorescence staining showed that the expression and cleavage of GSDMD were significantly increased in human intimal hyperplasia (Figs. 6A, B and S6A, B). Reversely, the expression and cleavage of GSDMD in wire*-*injured mouse femoral arteries were significantly attenuated in the circACTA2^+/+^ mice relative to the WT mice (Figs. 6C, D and S6C, D). Then, we overexpressed circACTA2 in VSMCs and evaluated the effect of circACTA2 overexpression on TNF-α-induced pyroptosis. As expected, overexpression of circACTA2 obviously inhibited the expression and cleavage of GSDMD induced by TNF-α (Fig. 6E). Correspondingly, immunofluorescence staining also revealed that the fluorescence intensity of GSDMD staining was attenuated in circACTA2-overexpressing VSMCs (Figs. 6F and S6E, F). Moreover, we found that the number of apoptotic-like cells and pyroptotic cells induced by TNF-α was significantly less in circACTA2-overexpressing VSMCs than in empty vector-transfected cells, as shown by PI staining (Figs. 6G and S6G). Finally, we explored the influence of the inhibition of inflammasome-mediated pyroptosis by circACTA2 overexpression on VSMC migration. The scratch wound-healing assay showed that overexpression of circACTA2 in VSMCs significantly suppressed TNF-α*-*induced migration in vitro (Fig. 6H, I). Likewise, lentivirus-mediated overexpression of circACTA2 (Fig. S6H) decreased TNF-α*-*induced invasion of VSMCs into Matrigel, as evidenced by Matrigel invasion assays (Fig. 6J, K). Taken together, these data support the idea that circACTA2 depresses VSMC inflammation and intimal hyperplasia via inhibiting NLRP3 inflammasome activation-mediated VSMC pyroptosis (Fig. 7).

## Discussion

Inflammation provides the essential signaling pathways that link hypertension, hypercholesterolemia, and vascular endothelial injury to the altered behavior of VSMCs, including VSMC abnormal proliferation, migration, and increased synthesis of extracellular matrix [40], and plays a crucial role in pathological vascular remodeling. Therefore, elucidation of the mechanisms underlying the regulation of inflammatory pathways could lead to development of new strategy to prevent and treat vascular remodeling-related cardiovascular diseases. Accumulating evidence has shown that inflammation is a complex pathophysiological process mediated by a variety of signaling molecules produced by leucocytes, macrophages and other cells, such as pro-inflammatory cytokines, cell adhesion molecules, and nuclear transcription factor-kB (NF-kB) [19, 21]. Recently, an increasing number of studies have revealed that non-coding RNAs, namely circRNAs, lncRNAs and miRNAs, are crucial regulators of many pathophysiological processes including inflammation [41, 42]. In particular, many circRNAs with important functions in the cardiovascular system are gradually being identified [43–46]. For example, circPTPRA is upregulated in serum of patients with atherosclerosis (As) promotes VSMC proliferation by sponging miR-636 and upregulating the transcription factor SP1 [47]. circANRIL has an atheroprotective effect by binding to pescadillo homologue 1 (PES1), an essential 60S-preribosomal assembly factor, and thus regulating ribosomal RNA (rRNA) maturation and resulting in the induction of apoptosis and inhibition of proliferation [48]. In addition, circRSF1 regulates ox-LDL-induced vascular endothelial cell proliferation, apoptosis, and inflammatory response by regulating the miR-135b-5p/HDAC1 axis [49]. Despite considerable progress elucidating the molecular regulation of VSMC proliferation and vascular inflammation by circRNAs, the functions of circRNAs in the pathogenesis of vascular remodeling remain to be fully elucidated due to the complexity of circRNA functions and the diversity of their structure circACTA2 is the first functional circRNA found in VSMCs by our group, which is formed through circularization of exon-5 to exon-9 of the Acta2 gene [29, 50]. We demonstrated that this circRNA promotes α-smooth muscle actin (α-SMA) expression via direct interaction with miR-548f-5p, functioning as a miRNA sponge to decrease miR-548f-5p repression of α-SMA gene (Acta2), and thus stabilizing actin filaments and enhancing contraction [29]. In this study, we found that circACTA2 was obviously down-regulated in human neointimal hyperplasia. Its overexpression in circACTA2 transgenic mice significantly decreased the neointimal hyperplasia of femoral arteries induced by wire injury. This suggests that circACTA2 exhibits an inhibitory effect on neointimal hyperplasia. Considering that inflammation is involved in neointimal hyperplasia and vascular remodeling, we assessed the relationship between circACTA2 anti-hyperplasia and anti-inflammation. Our data showed that levels of pro-inflammatory cytokines in the serum and neointimal hyperplasia of circACTA2 transgenic mice were significantly lower than those in wild-type mice. This suggests that circACTA2 suppresses wire injury-induced neointimal hyperplasia through reducing vascular inflammation.

The NLRP3 inflammasome activation has been known to exert a powerful pro-inflammatory effect via the release of IL-1β and IL-18 [16], and a number of diseases associated with NLRP3 inflammasome activation have been identified in humans [51]. In particular, the NLRP3 inflammasome activation plays an important role in the pathophysiology of cardiovascular disease. In the context of atherosclerosis and coronary artery disease, the expression level of NLRP3 in peripheral blood mononuclear cells (PBMCs) and arterial vascular tissue was positively correlated with the severity of the disease [52]. However, it remains to be determined whether the anti-inflammatory effect of circACTA2 is associated with its inhibition of NLRP3 inflammasome. As expected, the expression level of NLRP3 inflammasome components NLRP3, ASC, and procaspase-1 in the neointimal hyperplasia of circACTA2 transgenic mice was significantly lower than that of wild-type mice. Further, we overexpressed circACTA2 in mouse VSMCs by transfecting circACTA2 expression plasmids, and found that overexpression of circACTA2 significantly inhibited TNF-α-induced upregulation of NLRP3, ASC, procaspase-1, and subsequent release of IL-1β and IL-18, which is concomitant with a decrease in the activation of NLRP3 inflammasome. These results suggest that circACTA2 inhibits VSMC inflammation by suppressing the expression and activation of NLRP3 inflammasome. It has been known that some non-coding RNAs may contribute to disease progression by activating the NLRP3 inflammasome [53–56]. LncRNA GAS5 is down-regulated in cardiomyocytes with diabetic cardiomyopathy (DCM), and its overexpression suppresses the expression of NLRP3 and its downstream genes and caspase-1 activity, thus improving DCM [57]. miR-145 and miR-155 target the NLRP3 inflammasome by regulating upstream pathways of chronic inflammation in the context of atherosclerosis [58, 59]. LncRNA MEG3 acts as an endogenous sponge of miR-223 to increase the expression of NLRP3 and its related genes, thereby enhancing endothelial cell pyroptosis and atherosclerosis [60]. Compared with miRNAs and lncRNAs, the association of circRNAs with NLRP3 inflammasome activation has been poorly reported. Here, we confirmed that circACTA2 substantially inhibited the expression and activation of NLRP3 inflammasome in VSMCs exposed to pro-inflammatory environment, and its dysregulation was responsible for the development of vascular remodeling. This is in agreement with our previous study that circACTA2 maintained VSMC contractile phenotype by upregulating α-SMA expression and stabilizing actin filaments [29].

To further explore the molecular mechanism regarding how circACTA2 regulated the expression and activation of NLRP3 inflammasome, we investigated the effect of circACTA2 on the NF-κB signaling pathway, because NF-κB is known to play an important role in the regulation of NLRP3 [61, 62]. Our results clearly suggest that circACTA2 markedly attenuated TNF-α-induced expression and activation of NF-κB. Importantly, we found that circACTA2 specifically interacted with p50 or IκB, and demonstrated that the association of circACTA2 with p50 or IκB was direct, as shown by RNA pull-down assay using purified p50 and IκB. TNF-α treatment significantly reduced the interaction of circACTA2 with p50 or IκB. These findings suggest that circACTA2 may regulate the NLRP3 inflammasome by interacting with p50 and IκB. Upon TNF-α stimulation, IκB, the inhibitory subunit of NF-κB, undergoes phosphorylation and subsequent degradation, and p50 and p65 form heterodimers to synergistically enter the nucleus and transcriptionally activate downstream target genes [63]. Consistently, our results showed that overexpression of circACTA2 in VSMCs greatly decreased the nuclear translocation of p65 and p50 induced by TNF-α. However, circACTA2 could not prevent the degradation of IκB. Remarkably, a decoy oligonucleotide, which has the same sequence as the p50-binding site in the circACTA2 sequence, showed similar function with the full-sequence circACTA2 in binding to p50 and inhibiting the NLRP3 inflammasome. These findings strongly indicate that circACTA2 has the potential as a therapeutic target for vascular remodeling induced by NLRP3 inflammasome activation in the future. Yet, more rigorous investigations are required to test this possibility.

The NLRP3 inflammasome activation, which converts inactive pro-caspase-1 into active cleaved-caspase-1, then cleaves GSDMD into GSDMD-C and GSDMD-N, leads to pyroptosis and excessive inflammatory damage [64–67]. Although a few studies reported that circRNAs, such as circ_0138959, circ_003564, and circ-Katnal1, might have a regulatory role on GSDMD [68–70], circRNA regulation of GSDMD and pyroptosis has not been reported in cardiovascular diseases. Here, we explored the effect of circACTA2 on NLRP3 inflammasome-mediated pyroptosis in VSMCs. We found that cleaved GSDMD was significantly increased in the neointimal hyperplasia of both human and mouse arteries. Overexpression of circACTA2 in VSMCs largely attenuated the cleavage of GSDMD induced by TNF-α. Collectively, these data suggest that circACTA2 suppresses VSMC inflammation and intimal hyperplasia via inhibiting NLRP3 inflammasome activation and inflammasome-mediated pyroptosis.

In conclusion, circACTA2 inhibits vascular inflammation and neointimal hyperplasia through interacting with p50 and thus impeding the formation of the p50/p65 heterodimer and their nuclear translocation, resulting in the suppression of the expression and activation of NLRP3 inflammasome. The downregulation of circACTA2 expression in VSMCs contributes to vascular inflammation and remodeling. Targeting circACTA2 might be a potential therapeutic strategy to limit vascular inflammation and remodeling.

### Supplementary Information

Below is the link to the electronic supplementary material.

Supplementary file1 (PDF 1278 KB)

## Data Availability

All datasets generated and/or analyzed during this study are available from the corresponding author on reasonable request.
