# Supplementary material for: circACTA2 inhibits NLRP3 inflammasome-mediated inflammation via interacting with NF-κB in vascular smooth muscle cells
Source: Cell Mol Life Sci. 2023 Jul 27;80(8):229. doi: 10.1007/s00018-023-04840-6 (PMC10374705; doi:10.1007/s00018-023-04840-6)
Supplement: Supplementary file 1 — Supplementary file1 (PDF 1278 KB) [file 18_2023_4840_MOESM1_ESM.pdf]

circACTA2 inhibits NLRP3 inflammasome-mediated inflammation via interacting with NF-κB in vascular smooth muscle cells

Cellular and Molecular Life Sciences

Yang Bai[1], Long Zhang[1], Bin Zheng[1], Xinhua Zhang[1,5], Hong Zhang[2], Anning Zhao[3], Jing Yu[4], Zhan Yang[2], Jinkun Wen[1]

Correspondence should be addressed to Jinkun Wen (wjk@hebmu.edu.cn) and Zhan Yang (yangzhan@hebmu.edu.cn). Tel: 86311-8626-5563

Supplementary Table I. Primers for plasmid constructs

name	Sequences 5' to 3'
circACTA2-inf-F	CATTCCCTTTCTTTCCCTCAGCCGATCCAGACAGAGTATTTGCG
circACTA2-inf-R	CGTCTGCAGTTGATACTCACTGGCTCCATCCTGGCCTCTCTG

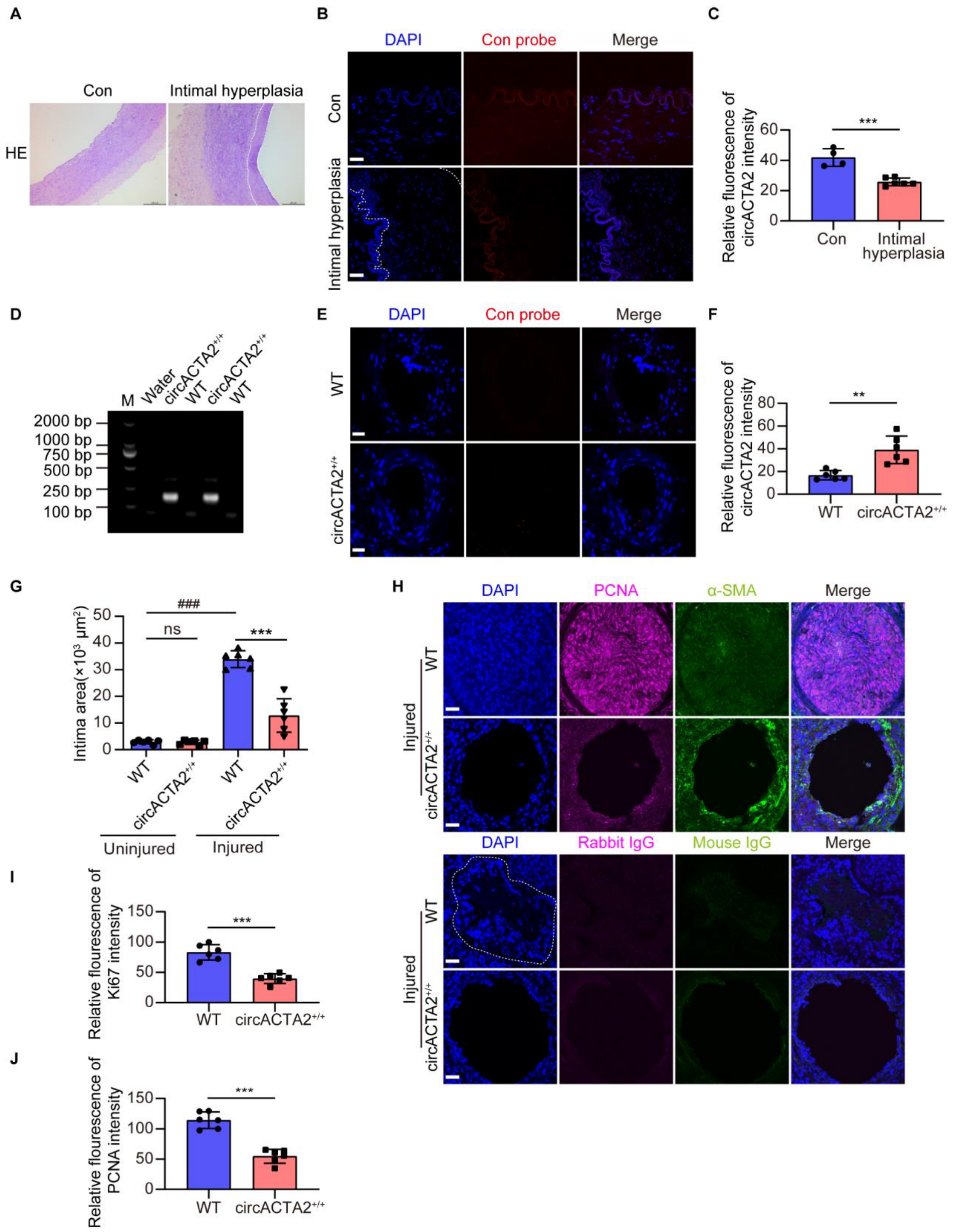

Fig. S1 circACTA2 is down-regulated in human intimal hyperplasia and its overexpression inhibits neointimal formation in mouse model of intimal hyperplasia. (**A**) Representative hematoxylin and eosin (HE)-stained cross-sections from the renal artery intimal hyperplasia of hypertensive patients, and neointimal hyperplasia was outlined by the dot line. Scale bars: 200 μm. (**B**) Con probe was

used to conduct RNA *in situ* hybridization in the renal artery intimal hyperplasia of hypertensive patients, and neointimal hyperplasia was outlined by the dot line. Scale bars: 30 μm. (**C**) Relative fluorescence intensity analysis of circACTA2 in Fig. 1A. ***$P < 0.001$ vs. Con. n=4 in Con group, n=6 in the patient group. (**D**) Genotype identification of circACTA2$^{+/+}$ mice by agarose gel electrophoresis. (**E**) Con probe was used to conduct RNA *in situ* hybridization in artery of WT and circACTA2$^{+/+}$ mice. (**F**) Relative fluorescence intensity analysis of circACTA2 in Fig. 1C. **$P <$ 0.01 vs. WT. n=6 per group. (**G**) Quantitative analysis of intimal area from uninjured and wire-injured arteries of WT and circACTA2$^{+/+}$ mice in Fig. 1E. ***$P < 0.001$ vs. WT; ###$P < 0.001$ vs. uninjured. n = 6 per group. (**H**) Co-immunofluorescence staining for PCNA (red), α-SMA (green), rabbit IgG (red), mouse IgG (green) and DAPI (blue) in injured arteries of WT and circACTA2$^{+/+}$ mice, and neointimal hyperplasia was outlined by the dot line. Scale bars: 30 μm. (**I**) Relative fluorescence intensity analysis of Ki67 in Fig. 1F. ***$P < 0.001$ vs. WT. n=6 per group. (**J**) Relative fluorescence intensity analysis of PCNA in Fig. S1H. ***$P < 0.001$ vs. WT. n=6 per group. Data are represented as mean ± SEM.

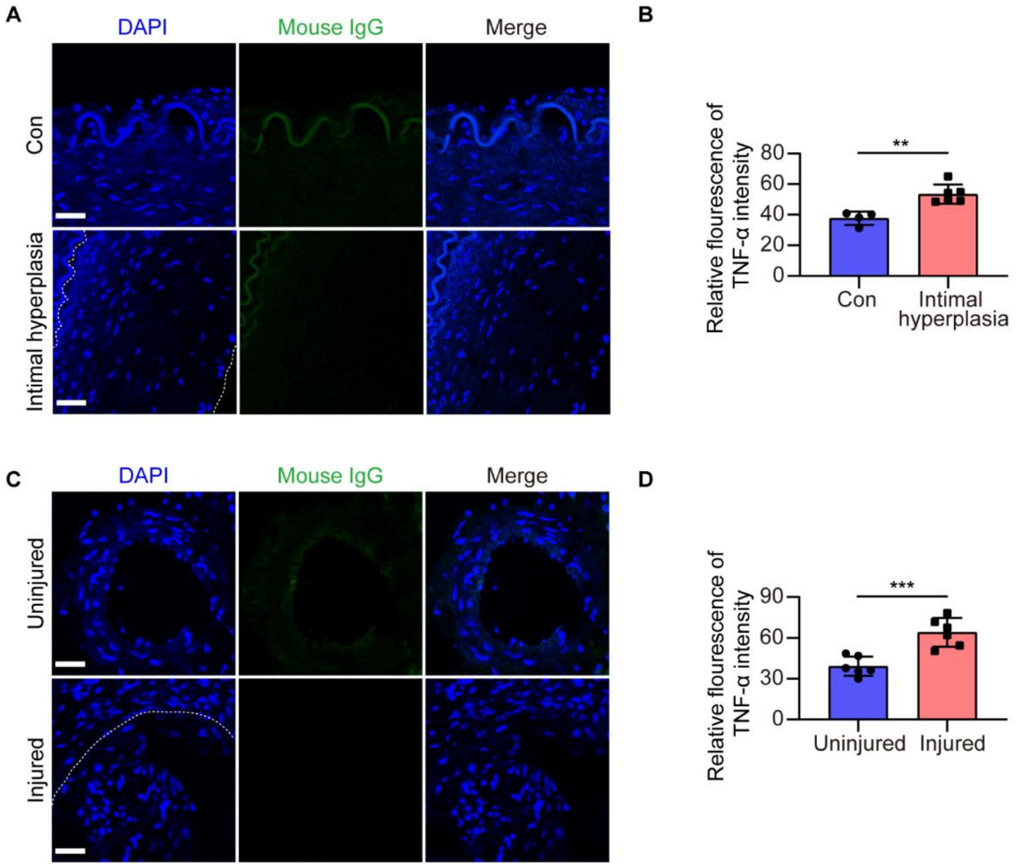

Fig. S2 circACTA2 attenuates the neointima formation by inhibiting inflammation. (**A**) Immunofluorescence staining of mouse IgG (green) and DAPI (blue) in the renal artery intimal hyperplasia of hypertensive patients, and neointimal hyperplasia was outlined by the dot line. Scale bars: 30 μm. (**B**) Relative fluorescence intensity analysis of TNF-α in Fig. 2A. ***P* < 0.01 vs. Con. n=4 in Con group, n=6 in the patient group. (**C**) Immunofluorescence staining of mouse IgG (green) and DAPI (blue) in uninjured and wire-injured arteries of WT mice, and neointimal hyperplasia was outlined by the dot line. Scale bars: 30 μm. (**D**) Relative fluorescence intensity analysis of TNF-α in Fig. 2B. ****P* < 0.001 vs. Con. n=6 per group. Data are represented as mean ± SEM.

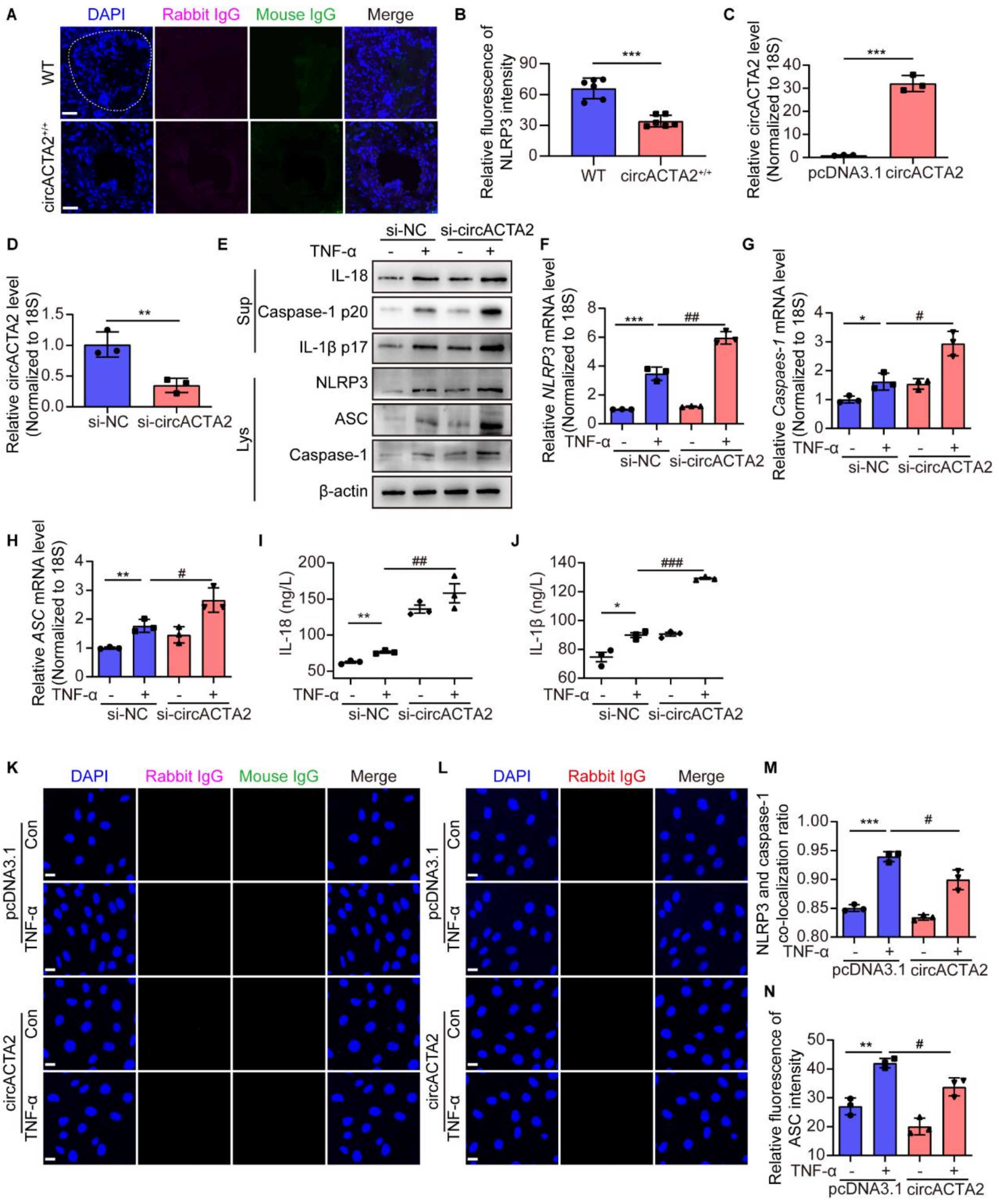

Fig. S3 circACTA2 alleviates VSMC inflammation by suppressing the activation of NLRP3 inflammasome. (**A**) Co-immunofluorescence staining for rabbit IgG (red), mouse IgG (green) and DAPI (blue) in injured arteries of WT and circACTA2$^{+/+}$ mice, and neointimal hyperplasia was outlined by the dot line. Scale bars: 30 μm. (**B**) Relative fluorescence intensity analysis of NLRP3 in Fig. 3A. ***P < 0.001 vs. Con. n=6 per group. (**C**) Overexpression efficiency of circACTA2 expressing plasmid detected by qRT-PCR in VSMCs. ***P < 0.001 vs. pcDNA3.1. n = 3 per group.

(**D**) Knockdown efficiency of si-circACTA2 detected by qRT-PCR in VSMCs. $^{**}P < 0.01$ vs. si-NC. n = 3 per group. (**E**) Western blotting detected the expression of IL-18, caspase-1 (p20), IL-1β (p17), NLRP3, caspase-1 and ASC in the supernatant and cellular lysates of circACTA2-silenced VSMCs, which were stimulated with or without TNF-α (25 ng/ml) for 24 h. (**F-G**) NLRP3 (**F**), caspase-1 (**G**) and ASC (**H**) mRNA expression detected by qRT-PCR in VSMCs treated as in (**E**). $^{*}P < 0.05$, $^{**}P < 0.01$, $^{***}P < 0.001$ vs. Control; $^{\#}P < 0.05$, $^{\#\#}P < 0.01$ vs. si-NC. n = 3 per group. (**I and J**) IL-18 (**I**) and IL-1β (**J**) content determined by ELISA in supernatant of VSMCs treated as in (**E**). $^{*}P < 0.05$, $^{**}P < 0.01$ vs. Control; $^{\#\#}P < 0.01$, $^{\#\#\#}P < 0.001$ vs. si-NC. n = 3 per group. (**K**) Co-immunofluorescence staining for rabbit IgG (red), mouse IgG (green) and DAPI (blue) in circACTA2-overexpressed VSMCs, which were stimulated with or without TNF-α (25 ng/ml) for 24 h. (**L**) Immunofluorescence staining for rabbit IgG (red) and DAPI (blue) in VSMCs treated as in (**K**). Scale bars: 20 μm. (**M**) Co-localization ratio of NLRP3 and Caspase-1 in Fig. 3H. $^{***}P <$ 0.001 vs. control; $^{\#}P < 0.05$ vs. pcDNA3.1. n = 3 per group. (**N**) Relative fluorescence intensity analysis of ASC in Fig. 3I. $^{**}P < 0.01$ vs. control; $^{\#}P < 0.05$ vs. pcDNA3.1. n=3 per group. Data are represented as mean ± SEM.

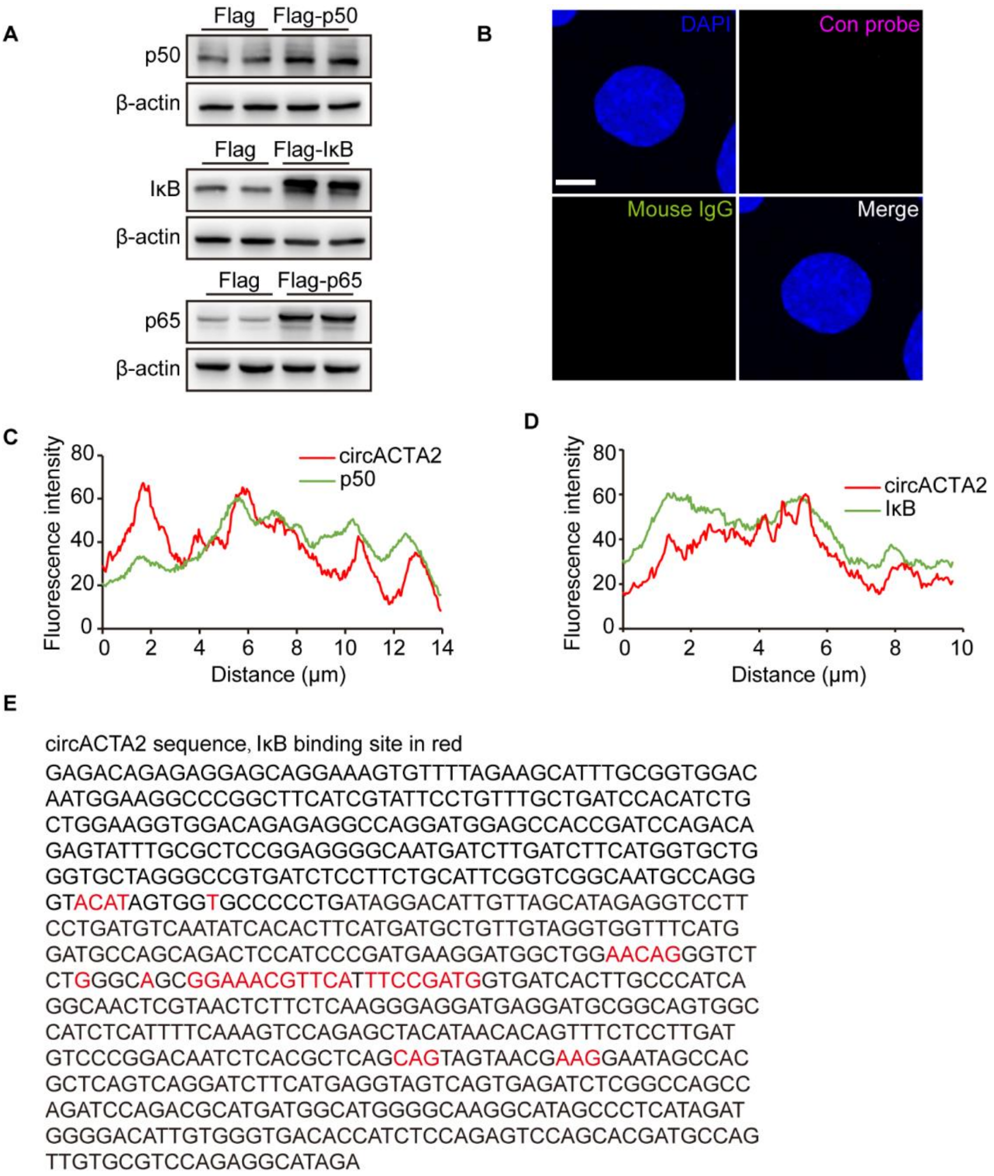

Fig. S4 circACTA2 inhibits the expression of NF-κB p65 and p50 subunits and interacts with them. (**A**) Western blotting detected the expression of p50, IκB and p65 in 293T cells transfected with p65, p50 and IκB expressing plasmids with Flag tag. (**B**) Co-immunofluorescence staining for Con probe (red), mouse IgG (green) and DAPI (blue) in VSMCs. Scale bars: 10 μm. (**C**) Fluorescence intensity analysis of circACTA2 and p50 in Fig. 4L. (**D**) Fluorescence intensity analysis of circACTA2 and IκB in Fig. 4M. (**E**) The interaction sites between circACTA2 and IκB were predicted by HDOCK website (http://hdock.phys.hust.edu.cn/).

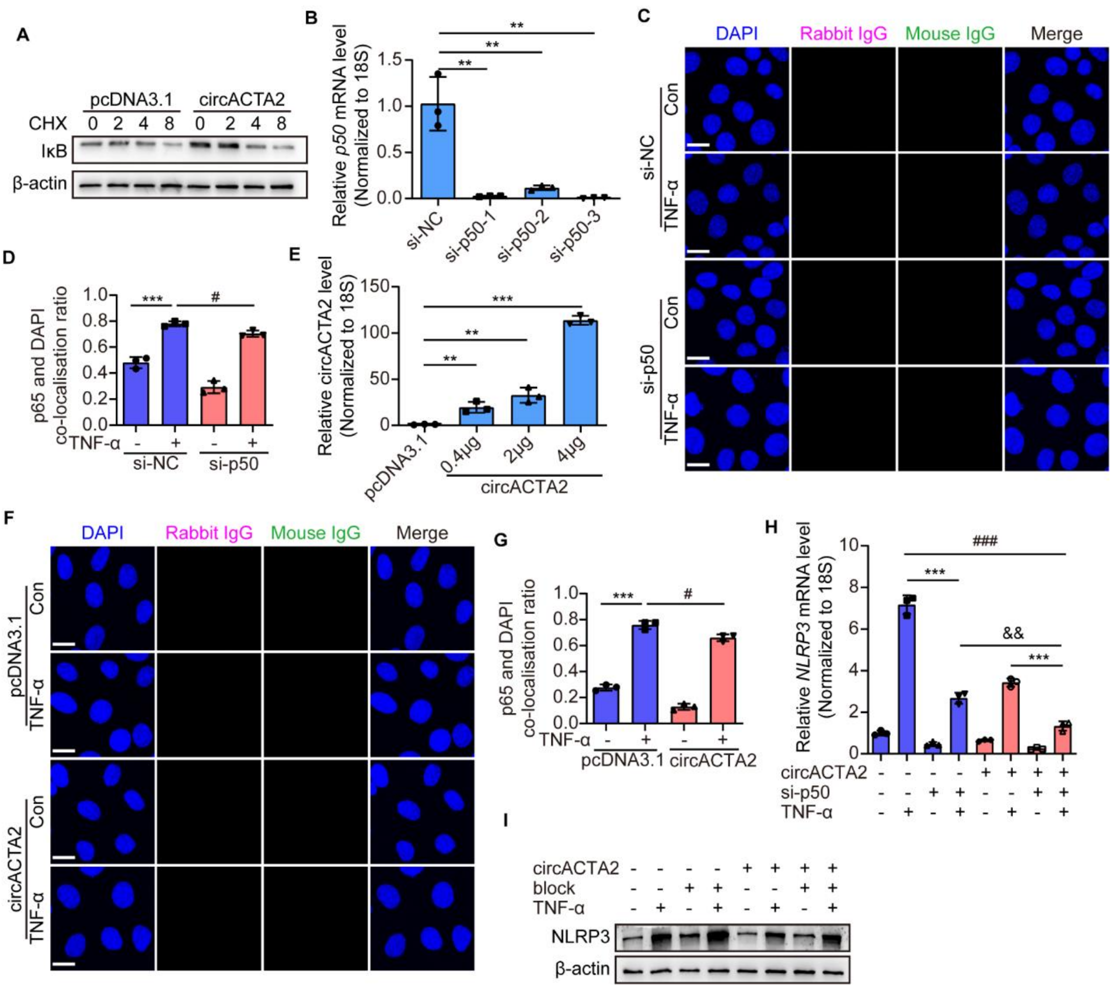

Fig. S5 circACTA2 impedes the p65 nuclear translocation induced by TNF-α via interacting with p50. (**A**) Western blot analysis detected the expression of IκB in circACTA2-overexpressed VSMCs, which were treated with CHX for different times. (**B**) VSMCs were transfected with 3 individual siRNAs targeting different regions of the p50 gene, and qRT-PCR detected p50 mRNA expression. ** $P < 0.01$ vs. si-NC. n = 3 per group. (**C**) Co-immunofluorescence staining for rabbit IgG (red), mouse IgG (green) and DAPI (blue) in VSMCs transfected with si-p50 and then stimulated with or without TNF-α for 30 min. Scale bars: 20 μm. (**D**) Co-localization ratio of p65 and DAPI in Fig. 5E. ***$P < 0.001$ vs. control; #$P < 0.05$ vs. si-NC. n = 3 per group. (**E**) qRT-PCR detected circACTA2 expression in VSMCs transfected with the different concentrations of circACTA2-expressing plasmid. **$P < 0.01$, ***$P < 0.001$ vs. pcDNA3.1. n = 3 per group. (**F**) Co-

immunofluorescence staining for rabbit IgG (red), mouse IgG (green) and DAPI (blue) in VSMCs transfected with circACTA2-expressing plasmid and then stimulated with or without TNF-α for 30 min. Scale bars: 20 μm. (**G**) Co-localization ratio of p65 and DAPI in Fig. 5G. ***P < 0.001 vs. control; #P < 0.05 vs. pcDNA3.1. n = 3 per group. (**H**) NLRP3 mRNA expression detected by qRT-PCR in p50-knocked down or/and circACTA2-overexpressed VSMCs, which were stimulated with or without TNF-α (25 ng/ml) for 24 h. ***P < 0.001 vs. si-NC, &&P < 0.01 vs. pcDNA3.1, ### P < 0.001 vs. si-NC and pcDNA3.1. n = 3 per group. (**I**) Western blot analysis detected the expression of NLRP3 in circACTA2-overexpressed VSMCs transfected with blocking oligo and stimulated with or without TNF-α (25 ng/ml) for 24 h.

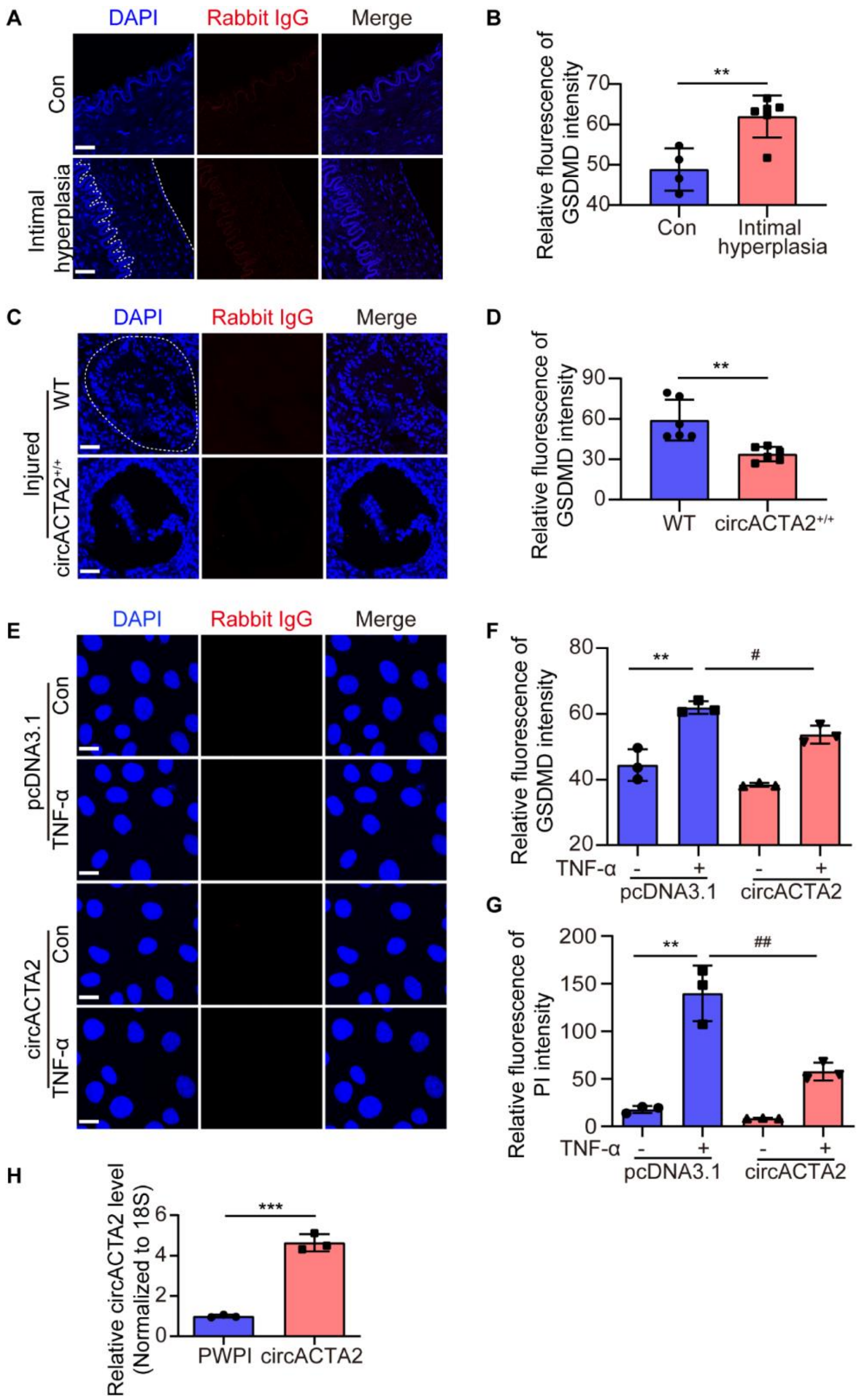

Fig. S6 circACTA2 alleviates inflammation through repressing NLRP3 inflammasome activation-mediated VSMC pyroptosis. (**A**) Immunofluorescence staining for rabbit IgG (red) and DAPI (blue) in the renal artery intimal hyperplasia of hypertensive patients, and neointimal hyperplasia was outlined by the dot line. Scale bars: 40 μm. (**B**) Relative fluorescence intensity analysis of GSDMD in Fig. 6B. ***P* < 0.01 vs. Con. n=4 in Con group, n=6 in the patient group. (**C**) Immunofluorescence

staining of rabbit IgG (red) and DAPI (blue) in injured arteries of WT and circACTA2$^{+/+}$ mice, and neointimal hyperplasia was outlined by the dot line. Scale bars: 40 μm. (**D**) Relative fluorescence intensity analysis of GSDMD in Fig. 6D. ***P* < 0.01 vs. WT. n=6 per group. (**E**) Immunofluorescence staining of rabbit IgG (red) and DAPI (blue) in circACTA2-overexpressed VSMCs stimulated with or without TNF-α (25 ng/ml) for 24 h. Scale bars: 15 μm. (**F)** Relative fluorescence intensity analysis of GSDMD in Fig. 6F. ***P* < 0.01 vs. control; #*P* < 0.05 vs. pcDNA3.1. n = 3 per group. (G) Relative fluorescence intensity analysis of PI in Fig. 6G. ***P* < 0.01 vs. control; ##*P* < 0.01 vs. pcDNA3.1. n = 3 per group. (**H**) circACTA2 expression detected by qRT-PCR in VSMCs infected with lentivirus carrying circACTA2. ****P* < 0.001 vs. PWPI. n = 3 per group. Data are represented as mean ± SEM.
